# Supplementary material for: High Levels of Mitotic Gene Conversion Are Needed to Effectively Purge Deleterious Mutations in Asexual Organisms
Source: Genome Biol Evol. 2026 Sep 8;18(11):evag224. doi: 10.1093/gbe/evag224 (PMC13767848; doi:10.1093/gbe/evag224)
Supplement: Supplementary file 1 [file evag224_supplementary_data.zip › R5 Supplementary text FINAL.docx]

**High levels of mitotic gene conversion are needed to effectively purge deleterious mutations in asexual organisms**

# Supplementary Text.

Dominik Kopčak, Matthew Hartfield

***Appendix 1:*** *Deriving single-locus results for the frequency of deleterious mutations and inbreeding coefficients.*

***Table S1:*** *Summary of model parameters and values used for simulations.*

***Figure S1:*** *Comparing analytical equations against exact numerical recursions, for varying rates of sex.*

***Figure S2:*** *Comparing analytical equations against exact numerical recursions, for varying rates of self-fertilisation.*

***Figure S3:*** *Mean fitness for different modes of uniparental reproduction.*

***Figure S4:*** *As Figure 4 in the main text, but with total count on a linear scale.*

***Figure S5:*** *Relative homozygosity as a function of uniparental reproduction rates for fixed s, h.*

***Figure S6:*** *Mean fitness for obligately asexual populations when mitotic gene conversion is present at different rates.*

***Figure S7:*** *Relative homozygosity for obligate asexual populations with different rates of mitotic gene conversion, for fixed s, h.*

***Figure S8****: As Figure 6 in the main text but with a population size of N = 1,000, and 𝜇, s increased 5-fold.*

***Figure S9****: As Figure 6 in the main text but with a population size of N = 10,000, and 𝜇, s increased 5-fold.*

***Figure S10:*** *Mean fitness for different modes of uniparental reproduction, when s, h are drawn from a distribution.*

***Figure S11:*** *Deleterious mutation counts under different uniparental reproductive modes, when s, h are drawn from a distribution.*

***Figure S12:*** *As Figure S11, but with the total mutation count on a linear scale.*

***Figure S13:*** *Relative homozygosity as a function of uniparental reproduction rates, when s, h are drawn from a distribution.*

***Figure S14:*** *Fitness measurements as a function of time when s, h are drawn from a distribution.*

***Figure S15:*** *Deleterious mutation counts for obligate asexual populations with mitotic GC present, when s, h are drawn from a distribution.*

***Figure S16:*** *Relative homozygosity for obligate asexual populations with different rates of mitotic gene conversion, when s, h are drawn from a distribution.*

***Figure S17:*** *As Figure 7 in the main text, but when s, h are drawn from a distribution.*

***Figure S18:*** *As figure S7 but when mitotic gene conversion rates are allowed to evolve.*

***Figure S19:*** *Fitness variance as a function of time, for fixed s, h values.*

***Figure S20:*** *Fitness variance as a function of time when s, h are drawn from a distribution.*

***Figure S21:*** *Fitness variance as a function of time, for fixed s, h values and when mitotic gene conversion is allowed to evolve.*

***Figure S22:*** *Comparing fitness measurements between the original WF model, and the new routine that implements GC into asexuals.*

***Figure S23:*** *Comparing mutation accumulation between the original WF model, and the new routine that implements GC into asexuals.*

***Figure S24:*** *Testing the evolving gene conversion simulation.*

# Appendix 1: Deriving single-locus results for the frequency of deleterious mutations and inbreeding coefficients

In the single-locus model, we assume a lifecycle consisting of selection, reproduction (either self-fertilisation or facultative sex), mutation, then mitotic gene conversion. We derive recursions for the frequency of the three genotypes (*aa*, *Aa*, and *AA* for *a* the wild-type mutations and *A* the derived, deleterious mutation), convert them to allele frequencies and inbreeding coefficients, then solve for steady-state values. We let *p_g_* denote the frequency of genotype *g* (e.g., *p_AA_* is the frequency of genotype *AA*).

***Selection.*** Let fitness for the three genotypes (*aa*, *Aa*, and *AA*), which we denote *W_g_*, be 1, 1–*hs* and 1–*s* respectively. The mean fitness equals W_m_ = *p_aa_* + *p_Aa_* (1–*hs*) + *p_AA_* (1–*s*). The change in frequency of genotype *g* over a single generation of selection is:

$$p_{g}^{sel}=\frac{p_{g}W_{g}}{W_{m}}$$

***Reproduction.*** We can partition reproduction into three outcomes; via outcrossing, via selfing, or via clonal reproduction. For outcrossing, we assume allele frequencies segregate independently. Hence, for example, the frequency of ancestral homozygotes after outcrossing is:

$$p_{aa}^{out}={{(p}_{aa}^{sel}+\frac{p_{Aa}^{sel}}{2})}^{2}$$

with a similar calculation for derived homozygotes *AA*. The frequency of heterozygotes after outcrossing is:

$$p_{Aa}^{out}=2(p_{aa}^{sel}+\frac{p_{Aa}^{sel}}{2})(p_{AA}^{sel}+\frac{p_{Aa}^{sel}}{2})$$

Under selfing, homozygotes will only produce homozygote offspring, while heterozygotes will produce heterozygotes with probability ½ and either of the homozygote with frequency ¼ each. This yields the recursion equation for the change in homozygotes following selfing (with the example given below for *aa* homozygotes):

$$p_{aa}^{self}=p_{aa}^{sel}+\frac{p_{Aa}^{sel}}{4}$$

The change in heterozygote frequency is:

$$p_{Aa}^{self}=\frac{p_{Aa}^{sel}}{2}$$

Under clonal reproduction, then genotype frequencies are unchanged between parent and offspring.

If self-fertilisation occurs with frequency *S*, then we can calculate the genotype frequencies after reproduction by weighing the contribution from outcrossing and selfing accordingly:

$$p_{g}^{S}=Sp_{g}^{self}+(1-S)p_{g}^{out}$$

Similarly, we can calculate the change in genotype frequencies following facultative sex, given a frequency of sex 𝜎 and clonal reproduction 1– 𝜎:

$$p_{g}^{A}=(1-\sigma)p_{g}+\sigma p_{g}^{out}$$

In subsequent sections we use $p_{g}^{R}$ to denote the allele frequency following any kind of reproduction.

***Mutation.*** We assume unidirectional mutation, i.e. allele *a* changes to *A* with probability 𝜇. Hence, *aa* mutates to *Aa* with probability 2 𝜇 (1– 𝜇), *aa* mutates to *AA* with probability 𝜇^2^, and *Aa* mutates to *AA* with probability 𝜇. This process yields the following recursions for $p_{g}^{M}$, the change in the frequency of genotype *g* after mutation:

$$p_{aa}^{M}=\left( 1-\mu\right)^{2}p_{aa}^{R}$$

$$p_{Aa}^{M}=2\mu\left( 1-\mu\right)p_{aa}^{R}+\left( 1-\mu\right)p_{Aa}^{R}$$

$$p_{AA}^{M}=p_{AA}^{R}+\mu^{2}p_{aa}^{R}+\mu p_{Aa}^{R}$$

***Mitotic gene conversion.*** Mitotic gene conversion acts with probability 𝛾. It only affects heterozygotes, and turns them into either homozygotes with probability 𝛾/2. This process yields the following recursions, which we denote $p_{g}^{GC}$:

$$p_{aa}^{GC}=p_{aa}^{M}+\frac{\gamma}{2}p_{Aa}^{M}$$

$$p_{Aa}^{GC}=(1-\gamma)p_{Aa}^{M}$$

$$p_{AA}^{GC}=p_{AA}^{M}+\frac{\gamma}{2}p_{Aa}^{M}$$

***Solving for steady-states.*** $p_{g}^{GC}$ denotes the final change in genotype frequency following one generation. We can use the change in genotype frequencies to derive the change in frequency for allele *A*, which equals $p_{A}^{GC}=p_{AA}^{GC}+p_{Aa}^{GC}/2$. We can also derive the change in inbreeding coefficient:

$$F^{GC}=\frac{p_{AA}^{GC}p_{aa}^{GC}-(p_{Aa}^{GC}/2)}{p_{A}^{GC}({1-p}_{A}^{GC})}$$

To then convert genotypes into allele frequencies, we can write each genotype in terms of allele frequencies, as described in the main text. For example, the frequency of *AA* can be written as *p_A_ +* *p_A_(1– p_A_)F*. These equations can be complicated, so we simplify them by taking a Taylor series assuming that the mutation rate $\mu$ and *p_A_* are both small (details are given in the Supplementary *Mathematica* file). From these simplified equations, we jointly solve for $p_{A}^{GC}-p_{A}=0$ and $F^{GC}-F=0$, yielding Equations 1–3 in the main text.

***Comparison with the model used by Flot* et al. *(2013)****.* There are some differences between the model used here and that employed by Flot *et al.* (2013). First, when modelling mutation we initially model the exact process, but subsequently linearise equations to remove terms of order $\mu^{2}$ and above. This linearisation is also employed by Flot *et al.* (2013). Second, we use gamma for the overall gene conversion rate, with a rate $\frac{\gamma}{2}$ that a heterozygote is converted into each homozygote. Conversely, Flot *et al.* (2013) use $\gamma$ to denote the conversion rate from heterozygote to each homozygote, so is effectively twice as large as what is used here. Finally, Flot *et al.* (2013) considered a stochastic model of mutation accumulation, while we use a deterministic model that does not take population-size effects into account.

**Table S1. Summary of model parameters and values used for simulations.** Note that for results with different population sizes (*N* equalling 1,000 and 10,000) the mutation rate and selection coefficients were scaled so *N𝜇, Ns* are the same as for *N* = 5,000 simulations.

| **Parameter** | **Values for simulations with f. e.*** | |
| --- | --- | --- |
|  | **constant** | **non-constant** |
| Genome size | Diploid, L = 25 Mbp | |
| Per-base mutation rate *𝜇* | $4\times{10}^{-9}$ | |
| Per-position recombination rate *r* | $4\times{10}^{-8}$ | |
| Population size *N* | 5 000 | |
| Sample size | 50 | |
| Selection | s = 0.01 | s ~ Gamma($\bar{s}$, $\alpha$)  mean $\bar{s}$ = 0.01  shape $\alpha$ = 0.2 |
| Dominance | h = 0.2 | h ~ U(0, $e^{-ks}$)  k = 91.63 |
| Mean gene conversion tract length | $\lambda$ = 4 000 bp | |
| Standard Deviation in gene conversion rate  (when it evolves) | $1\times{10}^{-8}$; $1\times{10}^{-7}$ | |

* f. e. - fitness effects; selection and dominance coefficients

| (a) $\hat{p}_{A}$: *h* = 0.01, *s* = 0.02   | (b) $\hat{F}$: *h* = 0.01, *s* = 0.02   |
| --- | --- |
| (c) $\hat{p}_{A}$: *h* = 0.5, *s* = 0.02   | (d) $\hat{F}$: *h* = 0.5, *s* = 0.02  |
| (e) $\hat{p}_{A}$: *h* = 0.01, *s* = 0.2 | (f) $\hat{F}$: *h* = 0.01, *s* = 0.2 |
| (g) $\hat{p}_{A}$: *h* = 0.5, *s* = 0.2 | (h) $\hat{F}$: *h* = 0.5, *s* = 0.2 |

***Figure S1: Comparing analytical equations against exact numerical recursions, for varying rates of sex.*** *As a function of the rate of gene conversion,* *(a), (c), (e), and (g) show the frequency of the deleterious mutation (Equation 1 in the main text), and (b), (d), (f) and (h) show the corresponding inbreeding coefficient F (Equation 2 in the main text). Closed circles gave values for the numerical recursions. Results are shown for different frequencies of asexual reproduction, as shown in the legend for panel (a). Other parameters are 𝜇 = 10^-8^, and in the recursions the initial frequency of genotype Aa is 0.001 and AA is zero, and recursions were run for 50,000 generations.*

| (a) $\hat{p}_{A}$: *h* = 0.01, *s* = 0.02   | (b) $\hat{F}$: *h* = 0.01, *s* = 0.02   |
| --- | --- |
| (c) $\hat{p}_{A}$: *h* = 0.5, *s* = 0.02   | (d) $\hat{F}$: *h* = 0.5, *s* = 0.02  |
| (e) $\hat{p}_{A}$: *h* = 0.01, *s* = 0.2 | (f) $\hat{F}$: *h* = 0.01, *s* = 0.2 |
| (g) $\hat{p}_{A}$: *h* = 0.5, *s* = 0.2 | (h) $\hat{F}$: *h* = 0.5, *s* = 0.2 |

***Figure S2: As Figure S1 but computed over different rates of self-fertilisation.*** *The plotted inbreeding coefficient is Equation 3 in the main text; closed circles give results from numerical recursions.*

| (a)   | (b)   |
| --- | --- |

***Figure S3: Mean fitness for different modes of uniparental reproduction.*** *Results are shown as a function of time, for (a) different rates of self-fertilisation or (b) asexual reproduction. Thick lines are mean values, while thinner shading around lines show 95% confidence intervals. Notice the change of scale on the y-axis between (a) and (b).*

***Figure S4: As Figure 4 in the main text, but with total count on a linear scale.***

 ***Figure S5: Relative homozygosity as a function of uniparental reproduction rates for fixed s, h.*** *Points are averages across all simulations. Error bars represent 95% confidence intervals; if they are not shown then they lie within a point.*

***Figure S6: Mean fitness for obligately asexual populations when mitotic gene conversion is present at different rates.*** *Results are shown as a function of time. Thick lines are mean values, while thinner shading around lines show 95% confidence intervals.*

***Figure S7: Relative homozygosity for obligate asexual populations with different rates of mitotic gene conversion, for fixed s, h.*** *Points are averages across all simulations. Error bars represent 95% confidence intervals; if they are not shown then they lie within a point.*

***Figure S8: As Figure 6 in the main text but with a population size of N = 1,000, and* 𝜇*, s increased 5-fold.***

***Figure S9: As Figure 6 in the main text but with a population size of N = 10,000, and* 𝜇*, s decreased 2-fold.***

| (a)   | (b)   |
| --- | --- |

***Figure S10: Mean fitness for different modes of uniparental reproduction, when s, h are drawn from a distribution.*** *Results are shown as a function of time, for (a) different rates of self-fertilisation or (b) asexual reproduction under the original WF model. Thick lines are mean values, while thinner shading around lines show 95% confidence intervals. Notice the change of scale on the y-axis between (a) and (b).*

***Figure S11: Deleterious mutation counts under different uniparental reproductive modes, when s, h are drawn from a distribution.*** *As a function of the rate of uniparental reproduction (either selfing or asexual reproduction), these plots show (top) the total mutation count present in the sample of 50 individuals, and (bottom) the number of homozygous genotypes present in the sample. Error bars represent 95% confidence intervals; if they are not shown then they lie within a point.*

***Figure S12: As Figure S11, but with the total mutation count on a linear scale.***

***Figure S13: Relative homozygosity as a function of uniparental reproduction rates, when s, h are drawn from a distribution.*** *Points are averages across all simulations. Error bars represent 95% confidence intervals; if they are not shown then they lie within a point.*

| (a)   | (b)   |
| --- | --- |

***Figure S14: Fitness measurements as a function of time when s, h are drawn from a distribution.*** *Shown are (a) mean fitness, and (b) variance in fitness for simulations of an asexual population subject to different levels of gene conversion. Thick lines are mean values, while thinner shading around lines show 95% confidence intervals.*

***Figure S15: Deleterious mutation counts for obligate asexual populations with mitotic GC present, when s, h are drawn from a distribution.*** *Results are shown as a function of the rate of gene conversion initiation at a single site. Plots show (top) the total mutation count present in the sample of 50 individuals, and (bottom) the number of homozygous genotypes present in the sample. Error bars represent 95% confidence intervals; if they are not shown then they lie within a point.*

***Figure S16: Relative homozygosity for obligate asexual populations with different rates of mitotic gene conversion, when s, h are drawn from a distribution.*** *Points are averages across all simulations. Error bars represent 95% confidence intervals; if they are not shown then they lie within a point.*

***Figure S17: As Figure 7 in the main text, but when s, h are drawn from a distribution.***

***Figure S18: As figure S7 but when mitotic gene conversion rates are allowed to evolve.***

| (a)   | (b)   |
| --- | --- |
| (c)   |  |

***Figure S19: Fitness variance as a function of time, for fixed s, h values.*** *Results are for different levels of (a) self-fertilisation, (b) asexual reproduction for the original Wright-Fisher simulation, or (c) GC in the modified non-WF routine. Thick lines are mean values, while thinner shading around lines show 95% confidence intervals.*

| (a)   | (b)   |
| --- | --- |

***Figure S20: Fitness variance as a function of time when s, h are drawn from a distribution.*** *Results are for different levels of (a) self-fertilisation, (b) asexual reproduction for the original Wright-Fisher simulation. Thick lines are mean values, while thinner shading around lines show 95% confidence intervals.*

***Figure S21: Fitness variance as a function of time, for fixed s, h values and when mitotic gene conversion is allowed to evolve.*** *Thick lines are mean values, while thinner shading around lines show 95% confidence intervals.*

| (a)   | (b)   |
| --- | --- |

***Figure S22: Comparing fitness measurements between the original WF model, and the new routine that implements GC into asexuals.*** *Plots show either (a) mean fitness or (b) fitness variance as a function of time. They compare these measurements under complete cloning in the WF model, and in the new non-WF-based routine under low levels of GC. Plots show both the mean value and 95% CI, although the latter are too small to see.*

***Figure S23: Comparing mutation accumulation between the original WF model, and the new routine that implements GC into asexuals.*** *Plots show either (top) total mutation count, or (bottom) number of homozygous genotypes. Plots compare high levels of asexuality under the original WF model (red points), to results obtained under the new non-WF-based GC routine (blue points). Bars represent 95% confidence intervals; if they are not seen then they lie within the point. Note that the total mutation counts and homozygous genotype counts under complete cloning in the original WF simulations (red dots, left-hand panels) match up with those for the new non-WF routine when gene conversion is low (blue dots, right-hand panels), demonstrating the equivalence of the two routines.*

| (a)   | (b)   |
| --- | --- |

***Figure S24: Testing the evolving gene conversion simulation.*** *Results show (a) the mean fitness as a function of time, and (b) mutation counts as a function of the mean gene conversion rate. Bands, lines are 95% confidence intervals. Compare with Figures S6 and 6 in the main text, respectively.*
