# Supplementary material for: High Levels of Mitotic Gene Conversion Are Needed to Effectively Purge Deleterious Mutations in Asexual Organisms
Source: Genome Biol Evol. 2026 Sep 8;18(11):evag224. doi: 10.1093/gbe/evag224 (PMC13767848; doi:10.1093/gbe/evag224)
Supplement: Supplementary file 1 [file evag224_supplementary_data.zip › Supplementary_Mathematica_File.pdf]

High levels of mitotic gene conversion are needed to effectively purge deleterious mutations in asexual organisms

Supplementary Mathematica file

Section A: Single-locus model, mutation-selection balance under facultative sex and gene conversion

In[ ]:=
```
(* This function clears existing commands from memory *)
Clear["`*"];
```

Recursions for asex with gene conversion, and selfing

This is a simple model for change in deleterious allele frequencies at a single locus, if affected by partial sex, mutation and gene conversion.
Let a be the wild-type and A the deleterious site.
Denote gaa, gAa, gAA the frequencies of aa, Aa and AA genotypes.
Note that gaa + gAa + gAA = 1.

Change in frequencies by selection, assuming mutations are deleterious

In[ ]:=
```
(* Individual fitness defintions *)
Waa = 1;
WAa = 1 - h s;
WAA = 1 - s;
(* Mean fitness *)
Wm = gaa * Waa + gAa * WAa + gAA * WAA
```

Out[ ]=
```
gaa + gAA (1 - s) + gAa (1 - h s)
```

In[ ]:=
```
gaas = gaa * Waa / Wm;
gAas = gAa * WAa / Wm;
gAAs = gAA * WAA / Wm;
```

Checking post-selection frequencies sum to 1:

In[ ]:=
```
gaas + gAas + gAAs // Simplify
```

Out[ ]=
```
1
```

Reproduction *with selfing*

Via sex (outcrossing):

In[ ]:=
```
gaaO = (gaas + gAas/2)^2;
gAaO = 2 (gaas + gAas/2) (gAAs + gAas/2);
gAAO = (gAAs + gAas/2)^2;
```

Via selfing:

In[ ]:=
```
gaaS = gaas + gAas/4;
gAaS = gAas/2;
gAAS = gAAs + gAas/4;
```

Total reproductive frequencies, assuming a mix of outcrossing and selfing (where S = frequency of selfing):

In[ ]:=
```
gaaRS = (1 - S) gaaO + S gaaS;
gAaRS = (1 - S) gAaO + S gAaS;
gAARS = (1 - S) gAAO + S gAAS;
```

Checking if summing to one:

In[ ]:=
```
gaaRS + gAaRS + gAARS // Simplify
```

Out[ ]=
```
1
```

Reproduction *via facultative sex*

Via asex (clonal reproduction):

In[ ]:=
```
gaaA = gaas;
gAaA = gAas;
gAAA = gAAs;
```

Total reproductive frequencies, assuming a mix of sex and asex (let σ = frequency of sex):

```
In[ ]:= gaaRA = σ gaaO + (1 - σ) gaaA;
        gAaRA = σ gAaO + (1 - σ) gAaA;
        gAARA = σ gAAO + (1 - σ) gAAA;
```

Checking if summing to one:

```
In[ ]:= gaaRA + gAaRA + gAARA // Simplify
Out[ ]=
        1
```

Mutation

Assume unidirectional mutations, so a -> A with probability μ and no back-mutation probability.
Hence aa -> aA with probability $2\mu(1-\mu)$, aa -> AA with probability μ^2, and aA -> AA with probability μ.
This yields the following recursions:

```
In[ ]:= gaaMS = (1 - μ)^2 gaaRS;
        gAaMS = 2 μ (1 - μ) gaaRS + (1 - μ) gAaRS;
        gAAMS = gAARS + μ^2 gaaRS + μ gAaRS;
```

Checking if summing to one:

```
In[ ]:= gaaMS + gAaMS + gAAMS // Simplify
Out[ ]=
        1
```

Same but for facultative sex:

```
In[ ]:= gaaMA = (1 - μ)^2 gaaRA;
        gAaMA = 2 μ (1 - μ) gaaRA + (1 - μ) gAaRA;
        gAAMA = gAARA + μ^2 gaaRA + μ gAaRA;
In[ ]:= gaaMA + gAaMA + gAAMA // Simplify
Out[ ]=
        1
```

Gene conversion

Gene conversion acts with probability γ.
It only affects heterozygotes, and turns them into homozygotes with probability γ (each homozygote with probability 1/2).
First for selfing:

```
In[ ]:= gaaGCS = gaaMS + γ/2 gAaMS;
        gAaGCS = (1 - γ) gAaMS;
        gAAGCS = gAAMS + γ/2 gAaMS;
```

Checking if summing to one:

```
In[ ]:= gaaGCS + gAaGCS + gAAGCS // Simplify
Out[ ]=
        1
```

Next for facultative sex:

In[ ]:=
```
gaaGCA = gaaMA + γ/2 gAaMA;
gAaGCA = (1 - γ) gAaMA;
gAAGCA = gAAMA + γ/2 gAaMA;
```

In[ ]:=
```
gaaGCA + gAaGCA + gAAGCA // Simplify
```

Out[ ]=
```
1
```

Steady-state solutions

Analytical solutions

This function converts genotype frequencies to allele frequencies while also considering inbreeding (F):

In[ ]:=
```
GtoP = {gaa → (1 - pA)^2 + pA (1 - pA) F,
   gAa → 2 pA (1 - pA) (1 - F), gAA → pA^2 + pA (1 - pA) F}
```

Out[ ]=
$$\{gaa \to (1 - pA)^2 + F\,(1 - pA)\,pA,\ gAa \to 2\,(1 - F)\,(1 - pA)\,pA,\ gAA \to F\,(1 - pA)\,pA + pA^2\}$$

We look for steady-state frequencies of pA and F, assuming small μ and pA. That way, we can use a series expansion to simplify the recursions while capturing the main effects of the reproductive mode and gene conversion.

Here's the appropriate expansion for pA following a single generation, given partial selfing:

In[ ]:=
```
Series[gAAGCS + gAaGCS/2 /. GtoP /. {pA → pA * ξ, μ → μ * ξ}, {ξ, 0, 1}] // Simplify //
   Normal;
% /. ξ → 1 // Simplify
```

Out[ ]=
$$pA + F\,(-1 + h)\,pA\,s - h\,pA\,s + \mu$$

We obtain the same result under facultative sex:

In[ ]:=
```
Series[gAAGCA + gAaGCA/2 /. GtoP /. {pA → pA * ξ, μ → μ * ξ}, {ξ, 0, 1}] // Simplify //
   Normal;
% /. ξ → 1 // Simplify
```

Out[ ]=
$$pA + F\,(-1 + h)\,pA\,s - h\,pA\,s + \mu$$

This gives us a system of equations for the change in frequency of pA; denote it pA'.
Hence we can write a general solution for the steady-state frequency of the deleterious allele (i.e. pA' - pA = 0), given an inbreeding coefficient F:

In[ ]:=
```
Solve[pA + F (-1 + h) pA s - h pA s + μ - pA == 0, pA]
```

Out[ ]=
$$\left\{\left\{pA \to -\frac{\mu}{(-F - h + F\,h)\,s}\right\}\right\}$$

Rewriting:

$$\frac{\mu}{(F + h - F h)\ s}$$

This equation matches previous results for allele frequencies under non-random mating (e.g. Glémin 2003).

Below are the equations for change in F following a single generation, assuming first selfing, and subsequetly for facultative sex:

In[∘]:=
$$\text{Series}\left[\frac{\text{gAAGCS gaaGCS} - \left(\frac{\text{gAaGCS}}{2}\right)^2}{\left(\text{gAAGCS} + \frac{\text{gAaGCS}}{2}\right)\left(\text{gaaGCS} + \frac{\text{gAaGCS}}{2}\right)} \text{ /. GtoP /. } \{pA \to pA * \xi,\ \mu \to \mu * \xi\},\ \{\xi, 0, 0\}\right] \text{ // Simplify // Normal;}$$

% /. ξ → 1 // Simplify

Out[∘]=
$$\frac{-pA\ (1 + F + F\ (-2 + h)\ s - h\ s)\ S\ (-1 + \gamma) + 2\ pA\ (1 + F\ (-1 + h)\ s - h\ s)\ \gamma + 2\ \gamma\ \mu}{2\ (pA + F\ (-1 + h)\ pA\ s - h\ pA\ s + \mu)}$$

In[∘]:=
$$\text{Series}\left[\frac{\text{gAAGCA gaaGCA} - \left(\frac{\text{gAaGCA}}{2}\right)^2}{\left(\text{gAAGCA} + \frac{\text{gAaGCA}}{2}\right)\left(\text{gaaGCA} + \frac{\text{gAaGCA}}{2}\right)} \text{ /. GtoP /. } \{pA \to pA * \xi,\ \mu \to \mu * \xi\},\ \{\xi, 0, 0\}\right] \text{ // Simplify // Normal;}$$

% /. ξ → 1 // Simplify

Out[∘]=
$$\frac{\gamma\ (pA - h\ pA\ s + \mu) + F\ pA\ ((-1 + \gamma)\ (-1 + \sigma) + s\ (-1 + h\ \gamma + \sigma - \gamma\ \sigma))}{pA + F\ (-1 + h)\ pA\ s - h\ pA\ s + \mu}$$

Now we can simultaneously solve for pA’ - pA = 0 and F’ - F = 0 to find the inbreeding coefficient under each mating-system. First for selfing:

In[∘]:=
$$\text{Solve}\left[\left\{(pA + F\ (-1 + h)\ pA\ s - h\ pA\ s + \mu) - pA == 0,\ \left(\frac{-pA\ (1 + F + F\ (-2 + h)\ s - h\ s)\ S\ (-1 + \gamma) + 2\ pA\ (1 + F\ (-1 + h)\ s - h\ s)\ \gamma + 2\ \gamma\ \mu}{2\ (pA + F\ (-1 + h)\ pA\ s - h\ pA\ s + \mu)}\right) - F == 0\right\},\ \{pA, F\}\right] \text{ // Simplify}$$

Out[∘]=
$$\left\{\left\{pA \to -\frac{(2 + (1 + (-2 + h)\ s)\ S\ (-1 + \gamma))\ \mu}{s\ (S\ (-1 + \gamma) + h\ (2 + (-2 + s)\ S)\ (-1 + \gamma) - 2\ \gamma)},\ F \to \frac{(-1 + h\ s)\ S\ (-1 + \gamma) + 2\ \gamma}{2 + (1 + (-2 + h)\ s)\ S\ (-1 + \gamma)}\right\}\right\}$$

Rewriting the pA, F terms to look nicer:

In[∘]:=
$$\frac{(2 - (1 - (2 - h)\ s)\ S\ (1 - \gamma))\ \mu}{s\ (S\ (1 - \gamma) + h\ (2 - (2 - s)\ S)\ (1 - \gamma) + 2\ \gamma)} - \left\{-\frac{(2 + (1 + (-2 + h)\ s)\ S\ (-1 + \gamma))\ \mu}{s\ (S\ (-1 + \gamma) + h\ (2 + (-2 + s)\ S)\ (-1 + \gamma) - 2\ \gamma)}\right\} \text{ // Simplify}$$

Out[∘]=
{0}

In[∘]:=
$$\frac{(1-h\,s)\,S\,(1-\gamma)+2\,\gamma}{2-(1-(2-h)\,s)\,S\,(1-\gamma)}-\left\{\frac{(-1+h\,s)\,S\,(-1+\gamma)+2\,\gamma}{2+(1+(-2+h)\,s)\,S\,(-1+\gamma)}\right\}\text{ // Simplify}$$

Out[∘]=
{0}

Note we can also write pA as $\frac{\mu}{(F+h-F\,h)\,s}$ but with F equal to the solution above:

In[∘]:=
$$\frac{(2-(1-(2-h)\,s)\,S\,(1-\gamma))\,\mu}{s\,(S\,(1-\gamma)+h\,(2-(2-s)\,S)\,(1-\gamma)+2\,\gamma)}-\left\{\frac{\mu}{(F+h-F\,h)\,s}\text{ /. }F\to\frac{(1-h\,s)\,S\,(1-\gamma)+2\,\gamma}{2-(1-(2-h)\,s)\,S\,(1-\gamma)}\right\}\text{ // Simplify}$$

Out[∘]=
{0}

Next we find the steady-state values of pA, F under facultative sex:

In[∘]:=
$$\text{Solve}\left[\left\{(pA+F\,(-1+h)\,pA\,s-h\,pA\,s+\mu)-pA==0,\ \left(\frac{\gamma\,(pA-h\,pA\,s+\mu)+F\,pA\,((-1+\gamma)\,(-1+\sigma)+s\,(-1+h\,\gamma+\sigma-\gamma\,\sigma))}{pA+F\,(-1+h)\,pA\,s-h\,pA\,s+\mu}\right)-F==0\right\},\{pA,F\}\right]\text{ // Simplify}$$

Out[∘]=
$$\left\{\left\{pA\to\frac{\mu\,(\gamma+s\,(-1+\gamma)\,(-1+\sigma)+\sigma-\gamma\,\sigma)}{s\,(\gamma+h\,(-1+\gamma)\,(s\,(-1+\sigma)-\sigma))},\ F\to\frac{\gamma}{\gamma+s\,(-1+\gamma)\,(-1+\sigma)+\sigma-\gamma\,\sigma}\right\}\right\}$$

Rewriting the pA, F terms to look nicer:

In[∘]:=
$$\frac{\mu\,(\gamma+(1-\gamma)\,(s\,(1-\sigma)+\sigma))}{s\,(\gamma+h\,(1-\gamma)\,(s\,(1-\sigma)+\sigma))}-\left\{\frac{\mu\,(\gamma+s\,(-1+\gamma)\,(-1+\sigma)+\sigma-\gamma\,\sigma)}{s\,(\gamma+h\,(-1+\gamma)\,(s\,(-1+\sigma)-\sigma))}\right\}\text{ // Simplify}$$

Out[∘]=
{0}

In[∘]:=
$$\frac{\gamma}{\gamma+(1-\gamma)\,(s\,(1-\sigma)+\sigma)}-\left\{\frac{\gamma}{\gamma+s\,(-1+\gamma)\,(-1+\sigma)+\sigma-\gamma\,\sigma}\right\}\text{ // Simplify}$$

Out[∘]=
{0}

Here too we can write pA as $\frac{\mu}{(F+h-F\,h)\,s}$ but with F equal to the steady-state value:

In[∘]:=
$$\frac{\mu\,(\gamma+(1-\gamma)\,(s\,(1-\sigma)+\sigma))}{s\,(\gamma+h\,(1-\gamma)\,(s\,(1-\sigma)+\sigma))}-\left\{\frac{\mu}{(F+h-F\,h)\,s}\text{ /. }F\to\frac{\gamma}{\gamma+(1-\gamma)\,(s\,(1-\sigma)+\sigma)}\right\}\text{ // Simplify}$$

Out[∘]=
{0}

Defining numerical functions, for single loci and multiple loci

Below we define functions for pA:

In[∘]:=
$$\text{pAnumSelf}[\mu\_,s\_,h\_,S\_,\gamma\_]:=\frac{(2-(1-(2-h)\,s)\,S\,(1-\gamma))\,\mu}{s\,(S\,(1-\gamma)+h\,(2-(2-s)\,S)\,(1-\gamma)+2\,\gamma)}$$

$$\text{pAnumAsex}[\mu\_,s\_,h\_,\sigma\_,\gamma\_]:=\frac{\mu\,(\gamma+(1-\gamma)\,(s\,(1-\sigma)+\sigma))}{s\,(\gamma+h\,(1-\gamma)\,(s\,(1-\sigma)+\sigma))}$$

Writing F as functions:

In[∘]:=
```
FnumSelf[s_, S_, γ_, h_] := ((1 - h s) S (1 - γ) + 2 γ) / (2 - (1 - (2 - h) s) S (1 - γ))

FnumAsex[s_, σ_, γ_] := γ / (γ + (1 - γ) (s (1 - σ) + σ))
```

Contour plots for manuscript

In[∘]:=
```
SetDirectory[NotebookDirectory[]];
```

In[∘]:=
```
pAAsex = Labeled[Labeled[ContourPlot[{pAnumAsex[10^-8, 0.02, 0.01, σ, γ] / pAnumAsex[10^-8, 0.02, 0.01, 1, 0],
    pAnumAsex[10^-8, 0.02, 0.2, σ, γ] / pAnumAsex[10^-8, 0.02, 0.2, 1, 0], pAnumAsex[10^-8, 0.02, 0.5, σ, γ] / pAnumAsex[10^-8, 0.02, 0.5, 1, 0]},
   {σ, 0, 1}, {γ, 0, 0.1}, PlotLayout → {"Column", 3}, ContourStyle → Black,
   PlotLegends → BarLegend[{Automatic, {0.0, 1.0}}],
   FrameTicks → {{{0.00, 0.02, 0.04, 0.06, 0.08, 0.10}, None},
     {{0, 0.2, 0.4, 0.6, 0.8, 1.0}, None}}, ImagePadding → {{30, 5}, {30, 5}},
   LabelStyle → Directive[FontFamily → "Arial", FontSize → 14, FontColor → Black],
   Method → {Spacings → {30, 0}}, Mesh → None, PlotRange → All],
  {Text@TraditionalForm@Style["Frequency of Sex, σ", 16],
   Text@TraditionalForm@Style["Mitotic Gene Conversion, γ", 16], Text@
    TraditionalForm@Style["(a) h = 0.01                                        (b) h =
       0.2                                  (c) h = 0.5              ", 16]},
  {Bottom, Left, Top}, RotateLabel → True], Text@TraditionalForm@
   Style["Frequency of deleterious mutation p̂_A, scaled by μ/hs \n", 16], Top]
```

Out[∘]=

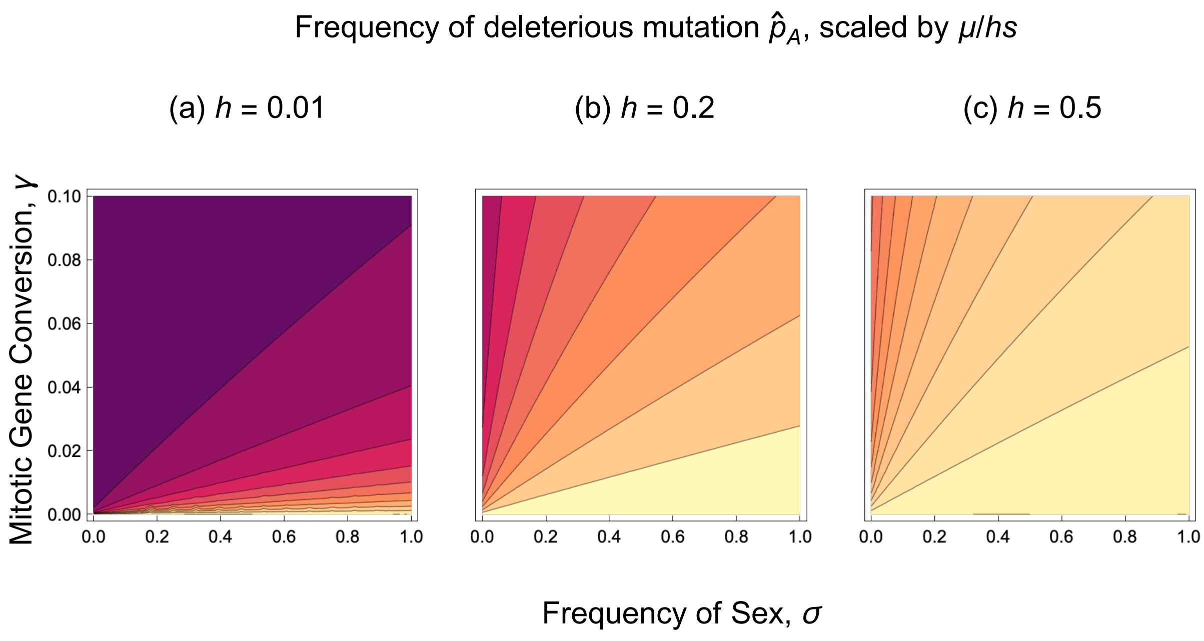

In[•]:=
```
pASelf =
  Labeled[ContourPlot[{pAnumSelf[10^-8, 0.02, 0.01, S, γ] / pAnumSelf[10^-8, 0.02, 0.01, 0, 0],
     pAnumSelf[10^-8, 0.02, 0.2, S, γ] / pAnumSelf[10^-8, 0.02, 0.2, 0, 0], pAnumSelf[10^-8, 0.02, 0.5, S, γ] / pAnumSelf[10^-8, 0.02, 0.5, 0, 0]},
    {S, 0, 1}, {γ, 0, 0.1}, PlotLayout → {"Column", 3}, ContourStyle → Black,
    PlotLegends → BarLegend[{Automatic, {0.0, 1.0}}],
    FrameTicks → {{{0.00, 0.02, 0.04, 0.06, 0.08, 0.10}, None},
      {{0, 0.2, 0.4, 0.6, 0.8, 1.0}, None}}, ImagePadding → {{30, 5}, {30, 5}},
    LabelStyle → Directive[FontFamily → "Arial", FontSize → 14, FontColor → Black],
    Method → {Spacings → {30, 0}}, Mesh → None, PlotRange → All],
   {Text@TraditionalForm@Style["Frequency of Selfing, S", 16],
    Text@TraditionalForm@Style["Mitotic Gene Conversion, γ", 16],
    Text@TraditionalForm@Style["(d) h = 0.01                                    (e) h
       = 0.2                              (f) h = 0.5             ",
      16]}, {Bottom, Left, Top}, RotateLabel → True]
```

Out[•]=

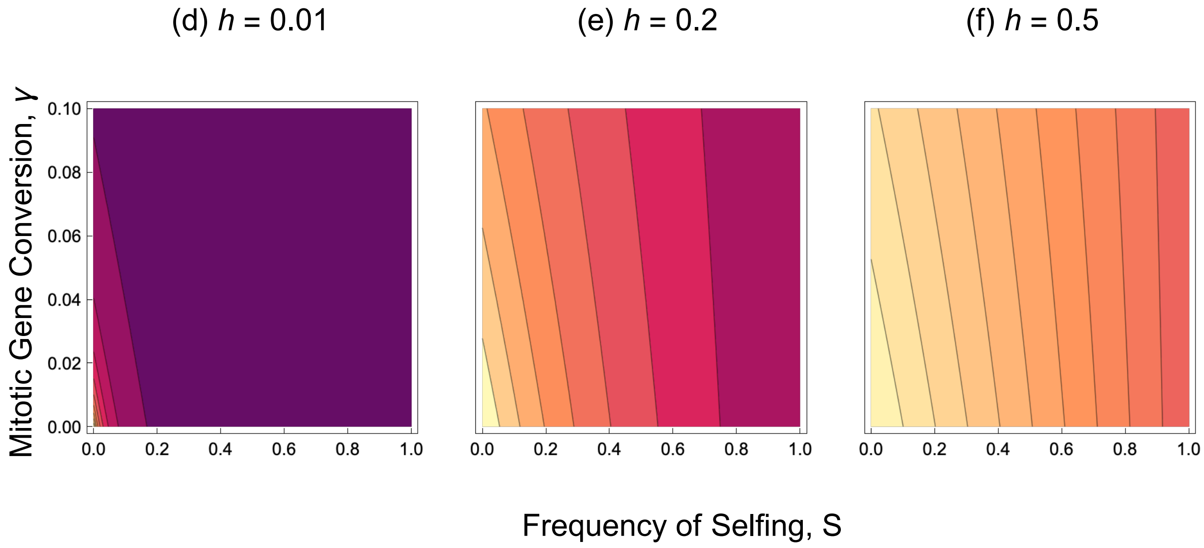

In[ ]:=
```
pAAll = Grid[{{pAAsex}, {pASelf}}]
```
Out[ ]=

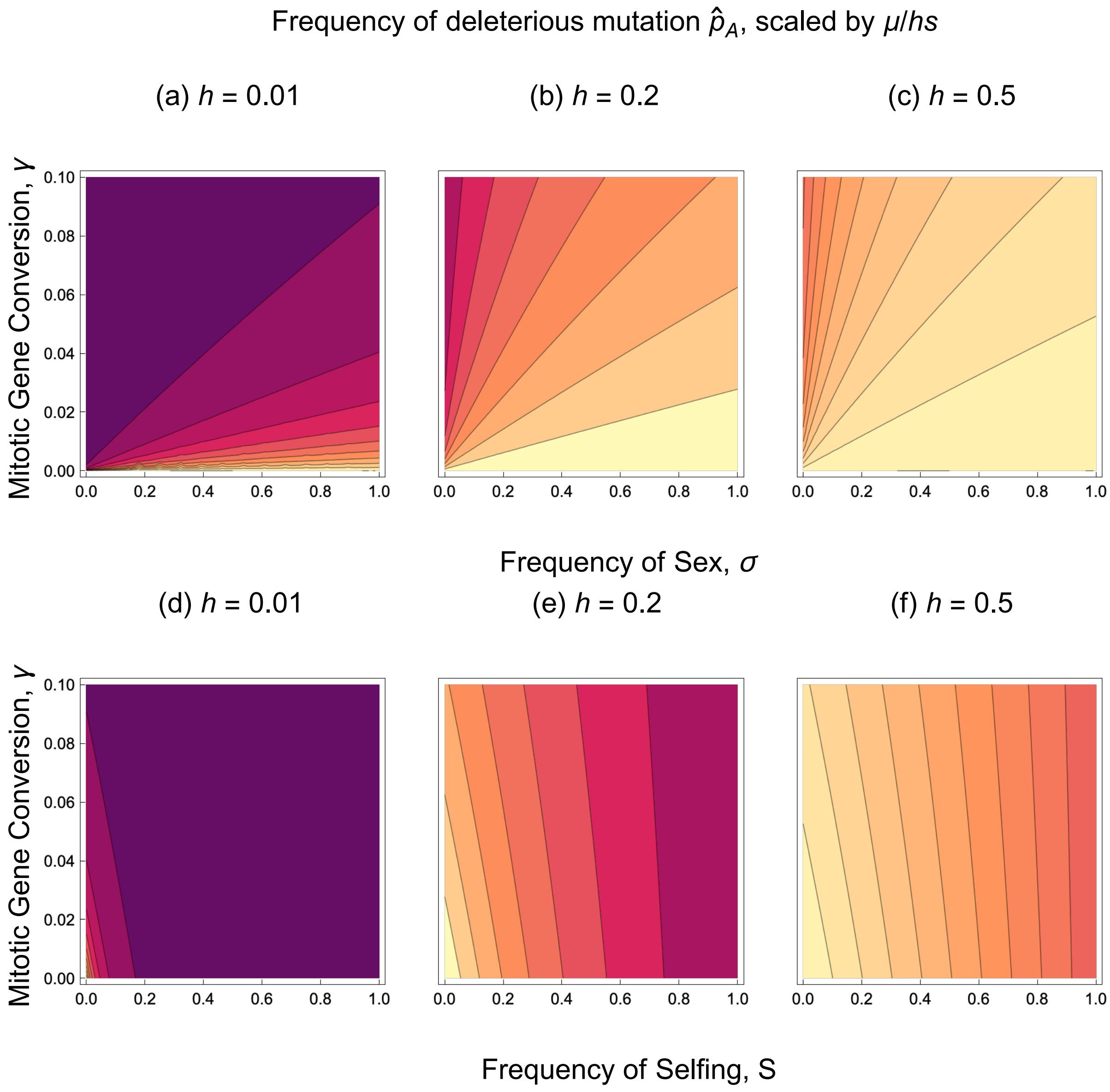

In[•]:=
```
pFAsex = Labeled[Labeled[ContourPlot[{FnumAsex[0.02, σ, γ], FnumAsex[0.2, σ, γ]},
    {σ, 0, 1}, {γ, 0, 0.1}, ContourStyle → Black, PlotLayout → {"Column", 2},
    PlotLegends → BarLegend[{Automatic, {0, 1.0}}],
    FrameTicks → {{{0.00, 0.02, 0.04, 0.06, 0.08, 0.10}, None},
      {{0, 0.2, 0.4, 0.6, 0.8, 1.0}, None}}, ImagePadding → {{30, 5}, {30, 5}},
    LabelStyle → Directive[FontFamily → "Arial", FontSize → 14, FontColor → Black],
    Method → {Spacings → {30, 0}}, PlotRange → All],
   {Text@TraditionalForm@Style["Frequency of Sex, σ           ", 16],
    Text@TraditionalForm@Style["Mitotic Gene Conversion, γ", 16],
    Text@TraditionalForm@
      Style["(a)  s = 0.02                                          (b)  s =
         0.2      ", 16]}, {Bottom, Left, Top}, RotateLabel → True],
  Text@TraditionalForm@Style["Reduction in Heterozygosity F̂", 16],
  Top]
```

Out[•]=

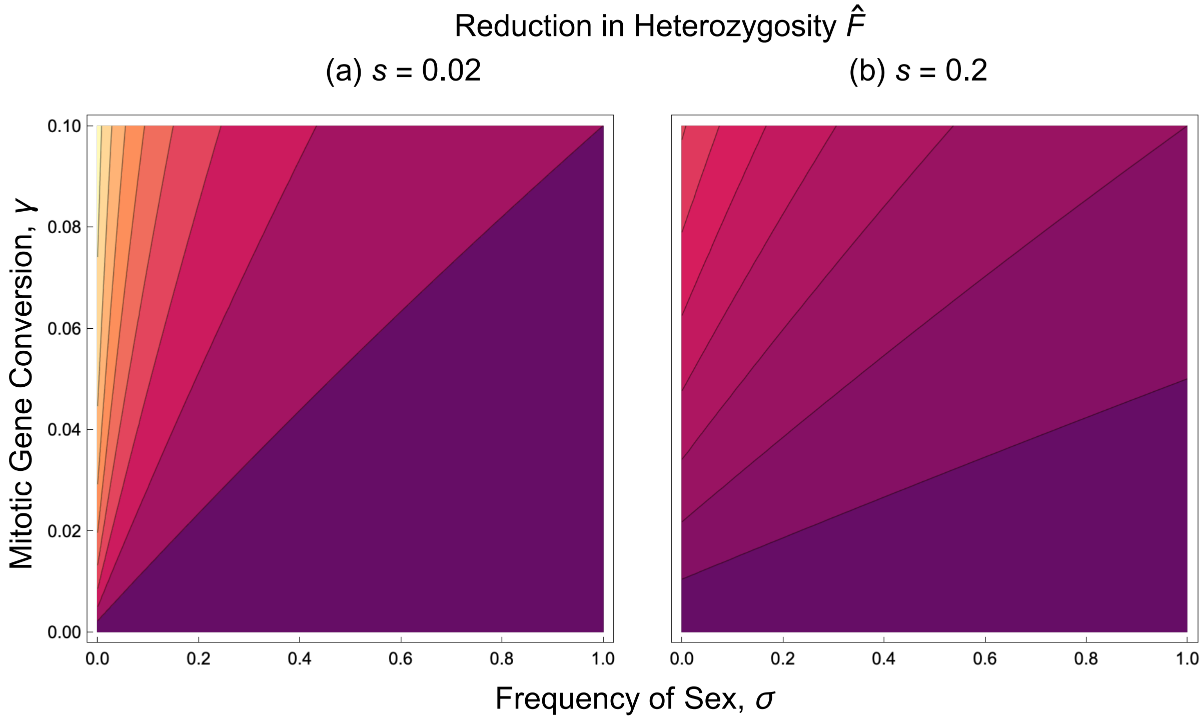

In[∘]:=
```
pFSelf = Labeled[ContourPlot[{FnumSelf[0.02, S, γ, 0.5], FnumSelf[0.2, S, γ, 0.5]},
   {S, 0, 1}, {γ, 0, 0.1}, ContourStyle → Black, PlotLayout → {"Column", 2},
   PlotLegends → BarLegend[{Automatic, {0, 1.0}}],
   FrameTicks → {{{0.00, 0.02, 0.04, 0.06, 0.08, 0.10}, None},
     {{0, 0.2, 0.4, 0.6, 0.8, 1.0}, None}}, ImagePadding → {{30, 5}, {30, 5}},
   LabelStyle → Directive[FontFamily → "Arial", FontSize → 14, FontColor → Black],
   Method → {Spacings → {30, 0}}, PlotRange → All],
  {Text@TraditionalForm@Style["Frequency of Selfing, S             ", 16],
   Text@TraditionalForm@Style["Mitotic Gene Conversion, γ", 16],
   Text@TraditionalForm@
     Style["(c)  s  = 0.02                                                    (d)  s
       = 0.2       ", 16]}, {Bottom, Left, Top}, RotateLabel → True]
```

Out[∘]=

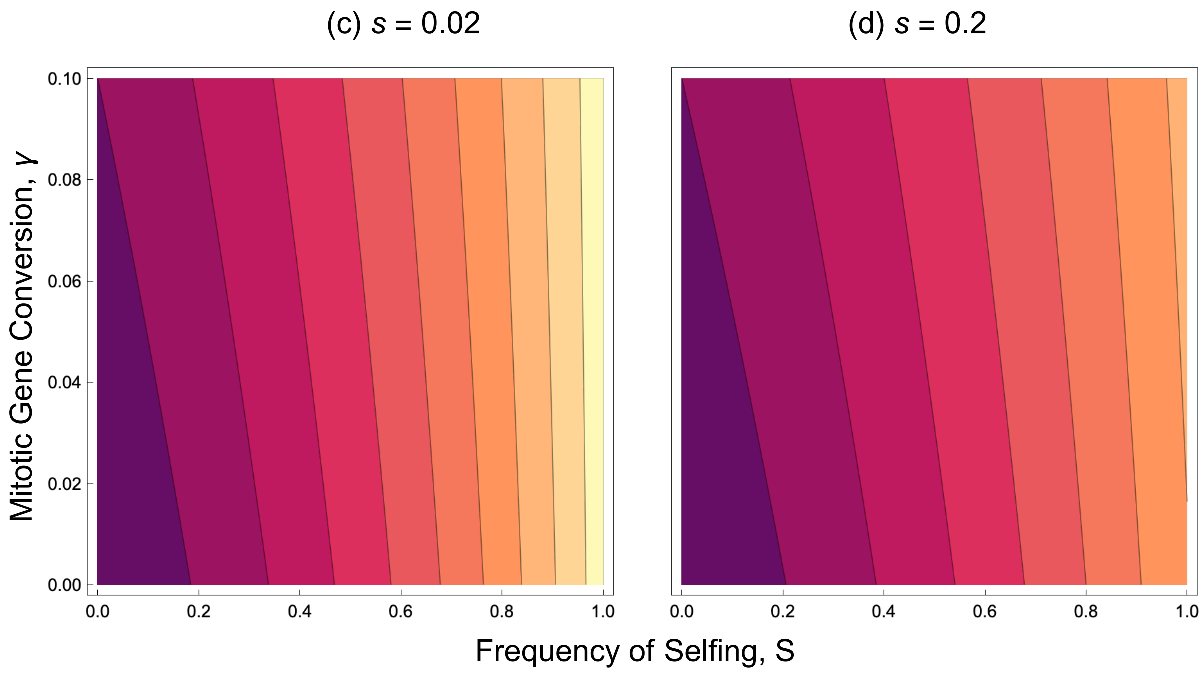

In[ ]:=
```
pFAll = Grid[{{pFAsex}, {pFSelf}}]
```
Out[ ]=

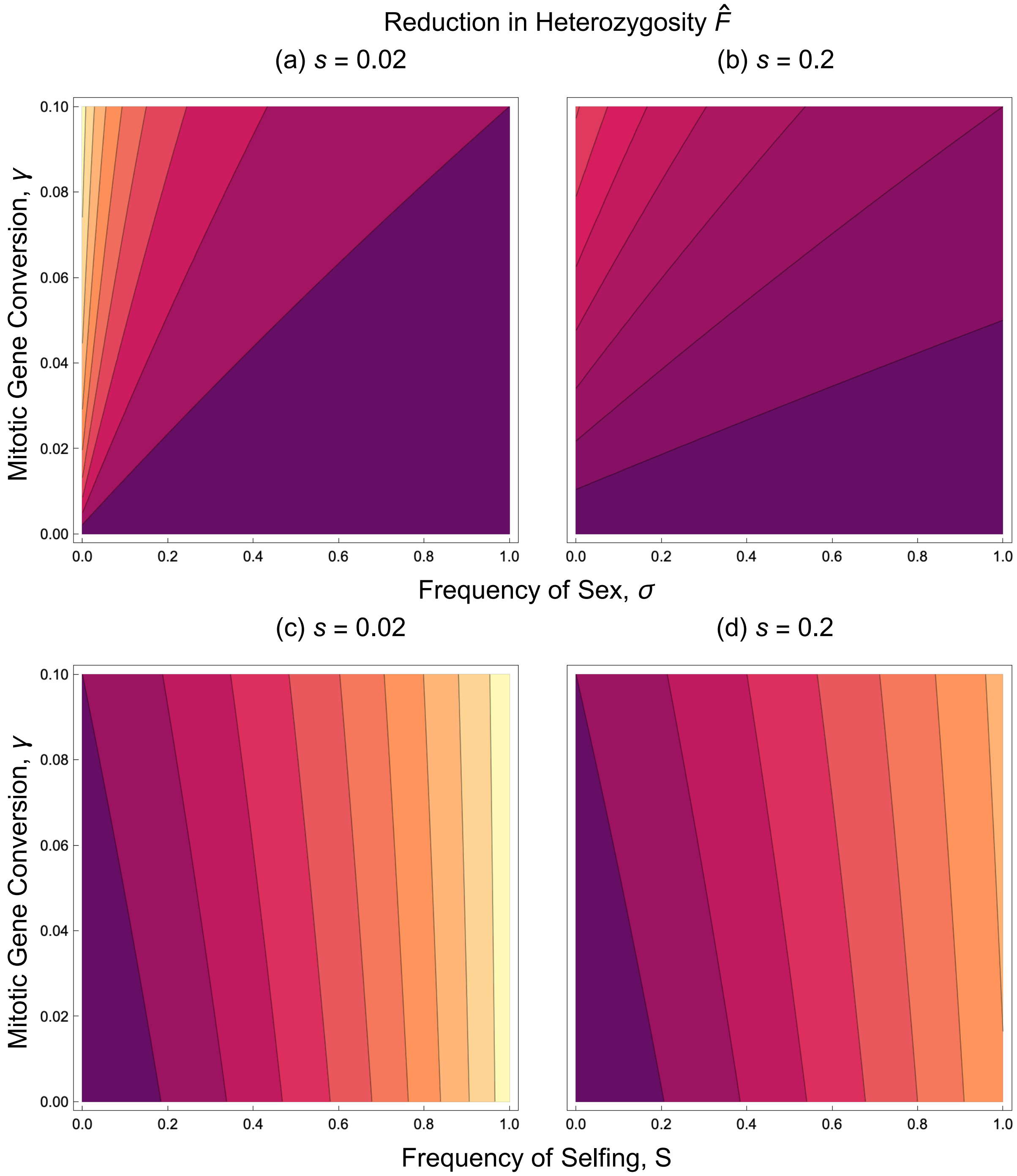


```
(* Extra command to remove tiling effects when exporting as PDF;
see https://mathematica.stackexchange.com/questions/2629/avoiding-
  white-lines-inside-filled-area-in-regionplot-exported-as-pdf-or-ps *)

pAAsex = pAAsex /. {EdgeForm[], r_?ColorQ, i___} :→ {EdgeForm[r], r, i};
Export["pAAsex_Plot.pdf", pAAsex, ImageResolution → 300];
Export["pAAsex_Plot.png", pAAsex, ImageResolution → 300];
```

```
pASelf = pASelf /. {EdgeForm[], r_?ColorQ, i___} :> {EdgeForm[r], r, i};
Export["pASelf_Plot.pdf", pASelf, ImageResolution → 300];
Export["pASelf_Plot.png", pASelf, ImageResolution → 300];
```

```
In[•]:= pAAll = pAAll /. {EdgeForm[], r_?ColorQ, i___} :> {EdgeForm[r], r, i};
Export["pAAll_Plot.pdf", pAAll, ImageResolution → 300];
Export["pAAll_Plot.png", pAAll, ImageResolution → 300];
```

```
pFAsex = pFAsex /. {EdgeForm[], r_?ColorQ, i___} :> {EdgeForm[r], r, i};
Export["FISAsex_plot.pdf", pFAsex, ImageResolution → 300];
Export["FISAsex_plot.png", pFAsex, ImageResolution → 300];
```

```
pFSelf = pFSelf /. {EdgeForm[], r_?ColorQ, i___} :> {EdgeForm[r], r, i};
Export["FISSelf_plot.pdf", pFSelf, ImageResolution → 300];
Export["FISSelf_plot.png", pFSelf, ImageResolution → 300];
```

```
In[•]:= pFAll = pFAll /. {EdgeForm[], r_?ColorQ, i___} :> {EdgeForm[r], r, i};
Export["FISAll_plot.pdf", pFAll, ImageResolution → 300];
Export["FISAll_plot.png", pFAll, ImageResolution → 300];
```

Numerical recursions, facultative sex

Below is the function for a change in genotype frequencies over a single generation:

```
In[•]:= RecurGCA[{gaa_, gAa_, gAA_}] :=
  {-(((-1 + μ) (4 gaa^2 (1 + (-1 + γ) μ) + gaa (-2 gAa (-1 + h s) (2 + γ - 2 μ + 2 γ μ) +
            4 gAA (-1 + s) (-1 + (-1 + γ) μ (-1 + σ) + σ - γ σ)) +
          gAa (-1 + h s) (2 gAA (-1 + s) γ + gAa (-1 + h s) (σ - μ σ + γ (2 + (-1 + μ) σ))))) /
      (4 (gaa + gAa + gAA - gAA s - gAa h s)^2)), ((-1 + γ) (-1 + μ)
      (4 gaa^2 μ + gaa (-2 gAa (-1 + h s) (1 + 2 μ) + 4 gAA (-1 + s) (μ (-1 + σ) - σ)) +
        gAa (-1 + h s) (2 gAA (-1 + s) + gAa (-1 + h s) (2 + (-1 + μ) σ)))) /
    (2 (gaa + gAa + gAA - gAA s - gAa h s)^2), (4 gAA^2 (-1 + s)^2 + 4 gaa^2 μ (γ + μ - γ μ) -
      2 gAa gAA (-1 + s) (-1 + h s) (γ (-1 + μ) - 2 (1 + μ)) +
      2 gaa gAa (-1 + h s) (-2 μ (1 + μ) + γ (-1 - μ + 2 μ^2)) -
      4 gaa gAA (-1 + s) (1 + γ (-1 + μ) (μ (-1 + σ) - σ) - μ^2 (-1 + σ) - σ + 2 μ σ) -
      gAa^2 (-1 + h s)^2 (2 μ (-2 + σ) - σ - μ^2 σ + γ (-1 + μ) (2 + (-1 + μ) σ))) /
    (4 (gaa + gAa + gAA - gAA s - gAa h s)^2)};
```

Defining range of γ, σ to be evaluated over:

```
In[•]:= γit = Table[0 + (0.1/10) j, {j, 0, 10}];
σit = {1, 0.5, 0.01};
```

h = 0.01, s = 0.02 plots

```
In[•]:= Clear[freqAa, μ, s, h, RecTime]
freqAa = 0.001;
μ = 10^-8;
s = 0.02;
h = 0.01;
RecTime = 50 000;
```

```
In[•]:= Clear[Vrin];
σ = σit⟦1⟧;
Vrin = Table[Clear[RecurRes, γ];
   γ = γit⟦j + 1⟧;
   RecurRes = NestList[RecurGCA, {1 - freqAa, freqAa, 0}, RecTime] // N;
   {γ, RecurRes⟦RecTime⟧⟦3⟧ + RecurRes⟦RecTime⟧⟦2⟧ / 2,
    (RecurRes⟦RecTime⟧⟦3⟧ * RecurRes⟦RecTime⟧⟦1⟧ - (RecurRes⟦RecTime⟧⟦2⟧ / 2)^2) /
     ((RecurRes⟦RecTime⟧⟦3⟧ + RecurRes⟦RecTime⟧⟦2⟧ / 2)
       (RecurRes⟦RecTime⟧⟦1⟧ + RecurRes⟦RecTime⟧⟦2⟧ / 2))}, {j, 0, 10}];
pArecσ1 = Partition[Drop[Flatten[Vrin], {3, Length[Flatten[Vrin]], 3}], 2];
Frecσ1 = Partition[Drop[Flatten[Vrin], {2, Length[Flatten[Vrin]] - 1, 3}], 2];

Clear[Vrin];
σ = σit⟦2⟧;
Vrin = Table[Clear[RecurRes, γ];
   γ = γit⟦j + 1⟧;
   RecurRes = NestList[RecurGCA, {1 - freqAa, freqAa, 0}, RecTime] // N;
   {γ, RecurRes⟦RecTime⟧⟦3⟧ + RecurRes⟦RecTime⟧⟦2⟧ / 2,
    (RecurRes⟦RecTime⟧⟦3⟧ * RecurRes⟦RecTime⟧⟦1⟧ - (RecurRes⟦RecTime⟧⟦2⟧ / 2)^2) /
     ((RecurRes⟦RecTime⟧⟦3⟧ + RecurRes⟦RecTime⟧⟦2⟧ / 2)
       (RecurRes⟦RecTime⟧⟦1⟧ + RecurRes⟦RecTime⟧⟦2⟧ / 2))}, {j, 0, 10}];
pArecσ05 = Partition[Drop[Flatten[Vrin], {3, Length[Flatten[Vrin]], 3}], 2];
Frecσ05 = Partition[Drop[Flatten[Vrin], {2, Length[Flatten[Vrin]] - 1, 3}], 2];

Clear[Vrin];
σ = σit⟦3⟧;
Vrin = Table[Clear[RecurRes, γ];
   γ = γit⟦j + 1⟧;
   RecurRes = NestList[RecurGCA, {1 - freqAa, freqAa, 0}, RecTime] // N;
   {γ, RecurRes⟦RecTime⟧⟦3⟧ + RecurRes⟦RecTime⟧⟦2⟧ / 2,
    (RecurRes⟦RecTime⟧⟦3⟧ * RecurRes⟦RecTime⟧⟦1⟧ - (RecurRes⟦RecTime⟧⟦2⟧ / 2)^2) /
     ((RecurRes⟦RecTime⟧⟦3⟧ + RecurRes⟦RecTime⟧⟦2⟧ / 2)
       (RecurRes⟦RecTime⟧⟦1⟧ + RecurRes⟦RecTime⟧⟦2⟧ / 2))}, {j, 0, 10}];
pArecσ001 = Partition[Drop[Flatten[Vrin], {3, Length[Flatten[Vrin]], 3}], 2];
Frecσ001 = Partition[Drop[Flatten[Vrin], {2, Length[Flatten[Vrin]] - 1, 3}], 2];
```

In[ ]:=
```
p1σ1 = ListPlot[pArecσ1, PlotStyle → {Red, PointSize[Large]}, PlotLegends →
    Placed[PointLegend[{Directive[Red, AbsolutePointSize[8]], Directive[Orange,
        AbsolutePointSize[8]], Directive[Blue, AbsolutePointSize[8]]},
      {"σ = 1", "σ = 0.5", "σ = 0.01"}], {0.4, 0.7}], LabelStyle → 18];
p1σ05 = ListPlot[pArecσ05, PlotStyle → {Orange, PointSize[Large]},
   PlotLegends → Placed[PointLegend[{Directive[Black, AbsolutePointSize[8]]},
      {"Numerical Recursions"}], {0.8, 0.8}], LabelStyle → 18];
p1σ001 = ListPlot[pArecσ001, PlotStyle → {Blue, PointSize[Large]}];

p2σ1 = Plot[{pAnumAsex[μ, s, h, 1, γin]},
   {γin, 0, 0.1}, PlotRange → Full, PlotStyle → {{Red}}, PlotLegends →
    Placed[LineLegend[{Black}, {"Equation 1"}, LabelStyle → 18], {0.75, 0.65}]];
p2σ05 = Plot[{pAnumAsex[μ, s, h, 0.5, γin]},
   {γin, 0, 0.1}, PlotRange → Full, PlotStyle → {{Orange}}];
p2σ001 = Plot[{pAnumAsex[μ, s, h, 0.01, γin]},
   {γin, 0, 0.1}, PlotRange → Full, PlotStyle → {{Blue}}];
```

In[ ]:=
```
plotpA1 = Labeled[Show[p1σ1, p2σ1, p1σ001, p2σ001, p1σ05, p2σ05, ImageSize → 550,
   LabelStyle → {FontFamily → "Arial", FontSize → 18, FontColor → Black}],
  {Text@TraditionalForm@Style["Frequency\nOf Deleterious\nAllele", 18], Text@
    TraditionalForm@Style["Mitotic Gene Conversion, γ", 18]}, {Left, Bottom}]
```

Out[ ]=

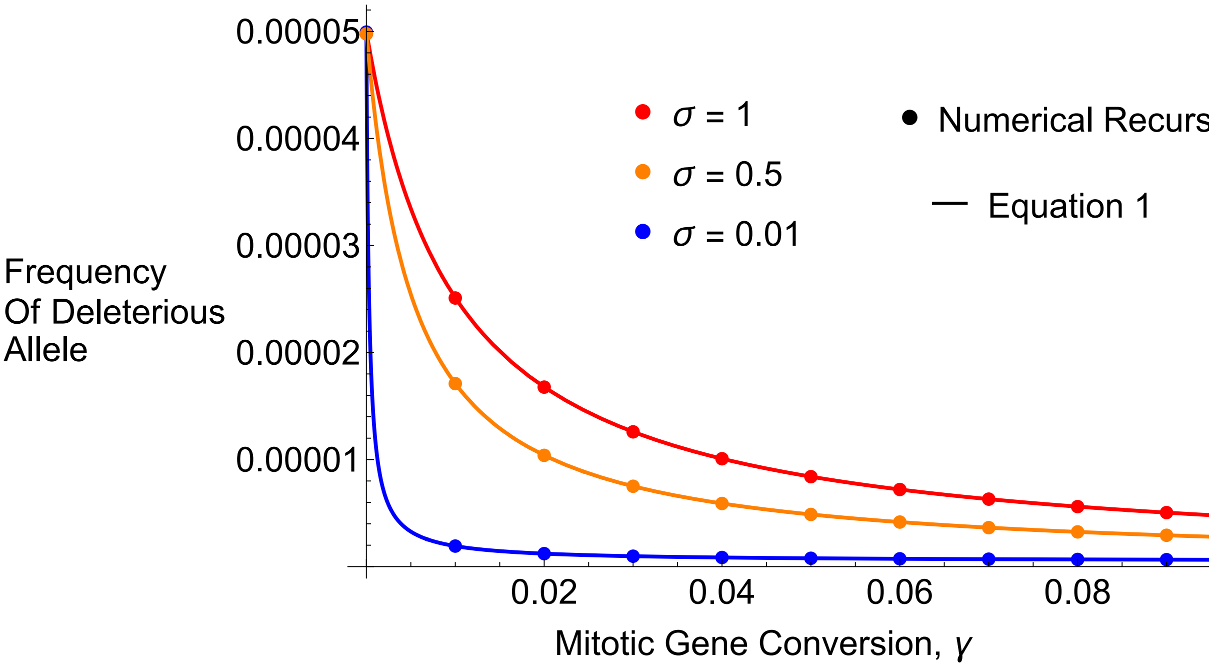


```
Export["RecPlotpA_h001_s002.pdf", plotpA1];
Export["RecPlotpA_h001_s002.jpg", plotpA1];
```

```
In[•]:= p1Fσ1 = ListPlot[Frecσ1, PlotStyle → {Red, PointSize[Large]}];
p1Fσ05 = ListPlot[Frecσ05, PlotStyle → {Orange, PointSize[Large]}];
p1Fσ001 = ListPlot[Frecσ001, PlotStyle → {Blue, PointSize[Large]}];

p2Fσ1 = Plot[{FnumAsex[s, 1, γin]}, {γin, 0, 0.1},
   PlotRange → Full, PlotStyle → {{Red}}, PlotLegends →
    Placed[LineLegend[{Black}, {"Equation 2"}, LabelStyle → 18], {0.75, 0.65}]];
p2Fσ05 = Plot[{FnumAsex[s, 0.5, γin]},
   {γin, 0, 0.1}, PlotRange → Full, PlotStyle → {{Orange}}];
p2Fσ001 = Plot[{FnumAsex[s, 0.01, γin]},
   {γin, 0, 0.1}, PlotRange → Full, PlotStyle → {{Blue}}];

In[•]:= plotF1 =
  Labeled[Show[p1Fσ001, p2Fσ001, p1Fσ1, p2Fσ1, p1Fσ05, p2Fσ05, ImageSize → 550,
    LabelStyle → {FontFamily → "Arial", FontSize → 18, FontColor → Black}],
   {Text@TraditionalForm@Style["Inbreeding\nCoefficient", 18], Text@
     TraditionalForm@Style["Mitotic Gene Conversion, γ", 18]}, {Left, Bottom}]

Out[•]=
```

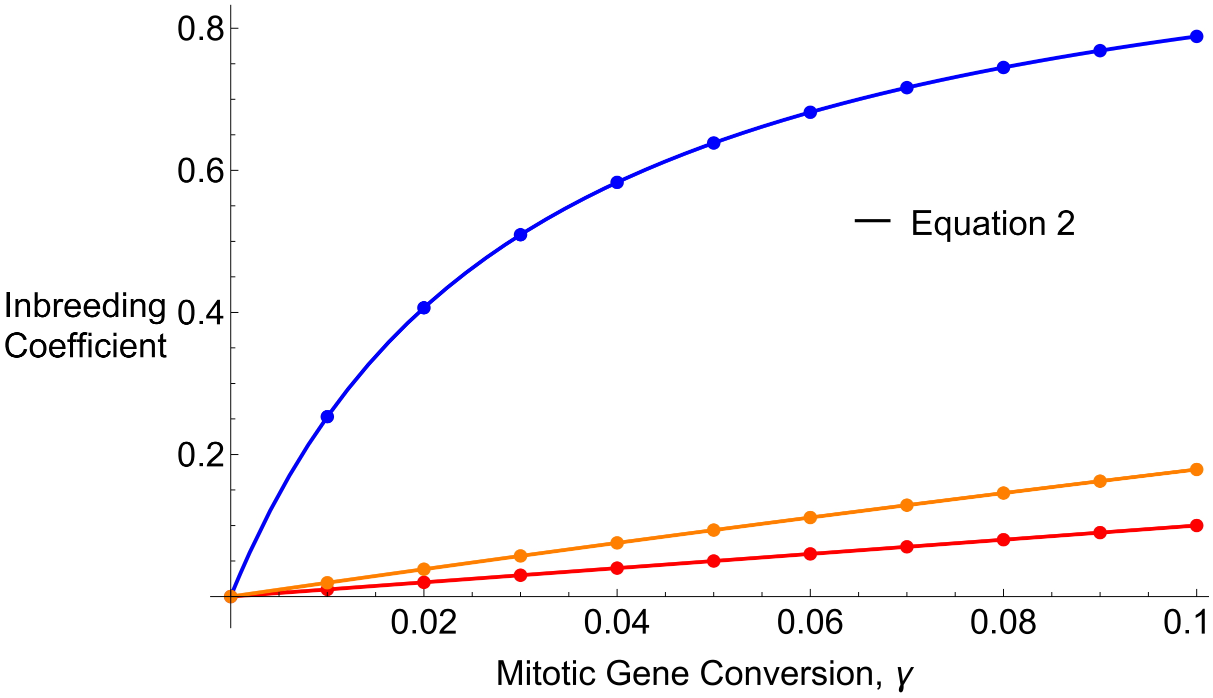


```
Export["RecPlotF_h001_s002.pdf", plotF1];
Export["RecPlotF_h001_s002.jpg", plotF1];
```

h = 0.5, s = 0.02 plots

```
In[•]:= Clear[freqAa, μ, s, h, RecTime]
freqAa = 0.001;
μ = 10^-8;
s = 0.02;
h = 0.5;
RecTime = 50 000;
```

In[ ]:=
```
Clear[Vrin];
σ = σit⟦1⟧;
Vrin = Table[Clear[RecurRes, γ];
   γ = γit⟦j + 1⟧;
   RecurRes = NestList[RecurGCA, {1 - freqAa, freqAa, 0}, RecTime] // N;
   {γ, RecurRes⟦RecTime⟧⟦3⟧ + RecurRes⟦RecTime⟧⟦2⟧ / 2,
    (RecurRes⟦RecTime⟧⟦3⟧ * RecurRes⟦RecTime⟧⟦1⟧ - (RecurRes⟦RecTime⟧⟦2⟧ / 2)^2) /
     ((RecurRes⟦RecTime⟧⟦3⟧ + RecurRes⟦RecTime⟧⟦2⟧ / 2)
       (RecurRes⟦RecTime⟧⟦1⟧ + RecurRes⟦RecTime⟧⟦2⟧ / 2))}, {j, 0, 10}];
pA2recσ1 = Partition[Drop[Flatten[Vrin], {3, Length[Flatten[Vrin]], 3}], 2];
F2recσ1 = Partition[Drop[Flatten[Vrin], {2, Length[Flatten[Vrin]] - 1, 3}], 2];

Clear[Vrin];
σ = σit⟦2⟧;
Vrin = Table[Clear[RecurRes, γ];
   γ = γit⟦j + 1⟧;
   RecurRes = NestList[RecurGCA, {1 - freqAa, freqAa, 0}, RecTime] // N;
   {γ, RecurRes⟦RecTime⟧⟦3⟧ + RecurRes⟦RecTime⟧⟦2⟧ / 2,
    (RecurRes⟦RecTime⟧⟦3⟧ * RecurRes⟦RecTime⟧⟦1⟧ - (RecurRes⟦RecTime⟧⟦2⟧ / 2)^2) /
     ((RecurRes⟦RecTime⟧⟦3⟧ + RecurRes⟦RecTime⟧⟦2⟧ / 2)
       (RecurRes⟦RecTime⟧⟦1⟧ + RecurRes⟦RecTime⟧⟦2⟧ / 2))}, {j, 0, 10}];
pA2recσ05 = Partition[Drop[Flatten[Vrin], {3, Length[Flatten[Vrin]], 3}], 2];
F2recσ05 = Partition[Drop[Flatten[Vrin], {2, Length[Flatten[Vrin]] - 1, 3}], 2];

Clear[Vrin];
σ = σit⟦3⟧;
Vrin = Table[Clear[RecurRes, γ];
   γ = γit⟦j + 1⟧;
   RecurRes = NestList[RecurGCA, {1 - freqAa, freqAa, 0}, RecTime] // N;
   {γ, RecurRes⟦RecTime⟧⟦3⟧ + RecurRes⟦RecTime⟧⟦2⟧ / 2,
    (RecurRes⟦RecTime⟧⟦3⟧ * RecurRes⟦RecTime⟧⟦1⟧ - (RecurRes⟦RecTime⟧⟦2⟧ / 2)^2) /
     ((RecurRes⟦RecTime⟧⟦3⟧ + RecurRes⟦RecTime⟧⟦2⟧ / 2)
       (RecurRes⟦RecTime⟧⟦1⟧ + RecurRes⟦RecTime⟧⟦2⟧ / 2))}, {j, 0, 10}];
pA2recσ001 = Partition[Drop[Flatten[Vrin], {3, Length[Flatten[Vrin]], 3}], 2];
F2recσ001 = Partition[Drop[Flatten[Vrin], {2, Length[Flatten[Vrin]] - 1, 3}], 2];
```

In[ ]:=
```
p3σ1 = ListPlot[pA2recσ1, PlotStyle → {Red, PointSize[Large]}];
p3σ05 = ListPlot[pA2recσ05, PlotStyle → {Orange, PointSize[Large]}];
p3σ001 = ListPlot[pA2recσ001, PlotStyle → {Blue, PointSize[Large]}];

p4σ1 = Plot[{pAnumAsex[μ, s, h, 1, γin]},
   {γin, 0, 0.1}, PlotRange → Full, PlotStyle → {{Red}}];
p4σ05 = Plot[{pAnumAsex[μ, s, h, 0.5, γin]},
   {γin, 0, 0.1}, PlotRange → Full, PlotStyle → {{Orange}}];
p4σ001 = Plot[{pAnumAsex[μ, s, h, 0.01, γin]},
   {γin, 0, 0.1}, PlotRange → Full, PlotStyle → {{Blue}}];
```

In[∘]:=
```
plotpA2 = Labeled[Show[p4σ001, p3σ001, p3σ05, p4σ05, p3σ1, p4σ1, ImageSize → 550,
    LabelStyle → {FontFamily → "Arial", FontSize → 18, FontColor → Black}],
  {Text@TraditionalForm@Style["Frequency\nOf Deleterious\nAllele", 18], Text@
    TraditionalForm@Style["Mitotic Gene Conversion, γ", 18]}, {Left, Bottom}]
```

Out[∘]=

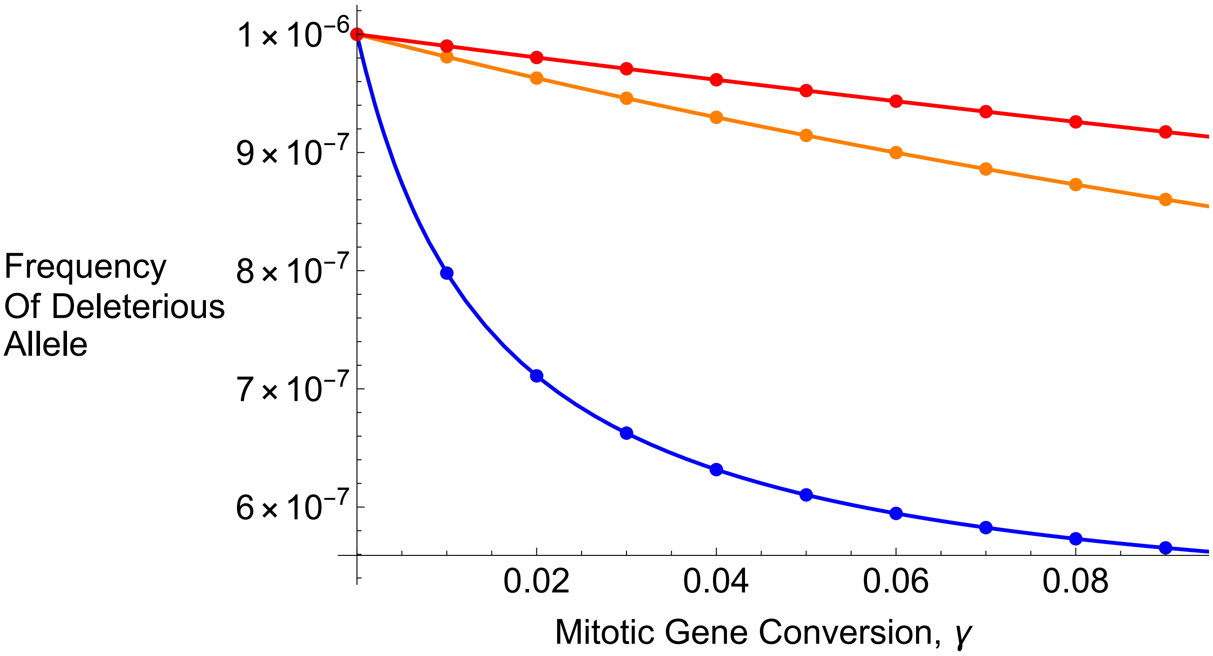

In[∘]:=
```
p3Fσ1 = ListPlot[F2recσ1, PlotStyle → {Red, PointSize[Large]}];
p3Fσ05 = ListPlot[F2recσ05, PlotStyle → {Orange, PointSize[Large]}];
p3Fσ001 = ListPlot[F2recσ001, PlotStyle → {Blue, PointSize[Large]}];

p4Fσ1 = Plot[{FnumAsex[s, 1, γin]},
   {γin, 0, 0.1}, PlotRange → Full, PlotStyle → {{Red}}];
p4Fσ05 = Plot[{FnumAsex[s, 0.5, γin]},
   {γin, 0, 0.1}, PlotRange → Full, PlotStyle → {{Orange}}];
p4Fσ001 = Plot[{FnumAsex[s, 0.01, γin]},
   {γin, 0, 0.1}, PlotRange → Full, PlotStyle → {{Blue}}];
```

In[ ]:=
```
plotF2 =
 Labeled[Show[p4Fσ001, p3Fσ001, p3Fσ1, p4Fσ1, p3Fσ05, p4Fσ05, ImageSize → 550,
   LabelStyle → {FontFamily → "Arial", FontSize → 18, FontColor → Black}],
  {Text@TraditionalForm@Style["Inbreeding\nCoefficient", 18], Text@
    TraditionalForm@Style["Mitotic Gene Conversion, γ", 18]}, {Left, Bottom}]
```

Out[ ]=

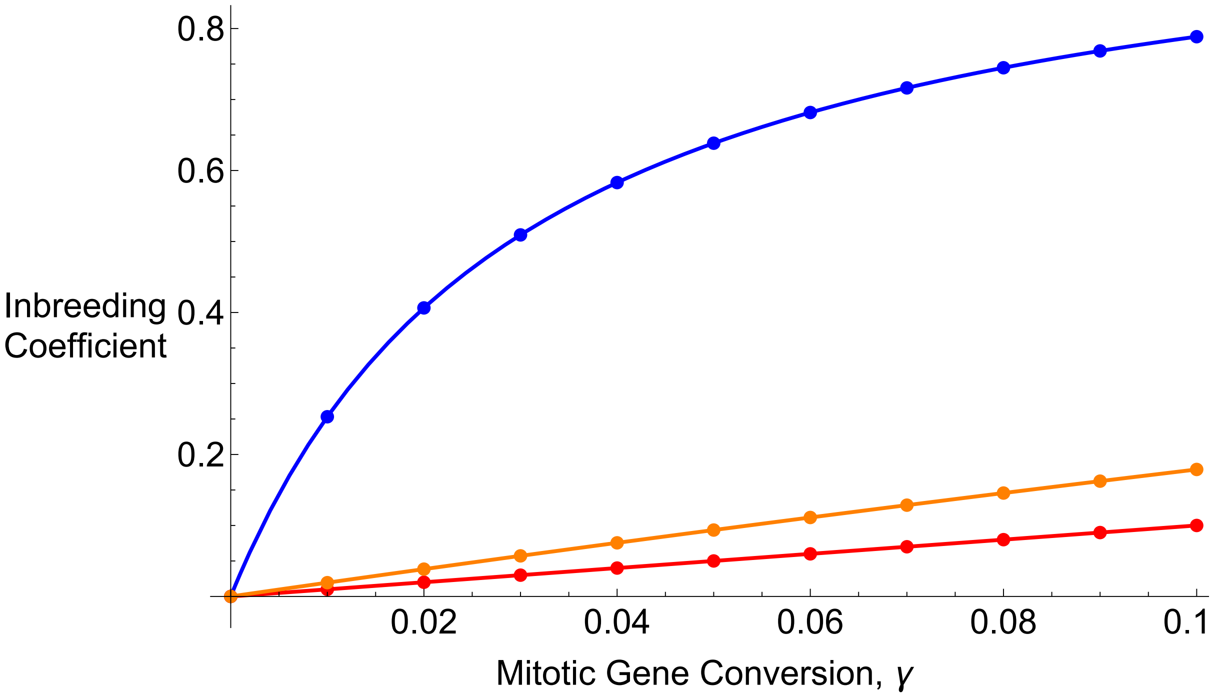


```
Export["RecPlotpA_h05_s002.pdf", plotpA2];
Export["RecPlotpA_h05_s002.jpg", plotpA2];

Export["RecPlotF_h05_s002.pdf", plotF2];
Export["RecPlotF_h05_s002.jpg", plotF2];
```

h = 0.01, s = 0.2 plots

In[ ]:=
```
Clear[freqAa, μ, s, h, RecTime]
freqAa = 0.001;
μ = 10^-8;
s = 0.2;
h = 0.01;
RecTime = 50 000;
```

In[ ]:=
```
Clear[Vrin];
σ = σit[[1]];
Vrin = Table[Clear[RecurRes, γ];
   γ = γit[[j + 1]];
   RecurRes = NestList[RecurGCA, {1 - freqAa, freqAa, 0}, RecTime] // N;
   {γ, RecurRes[[RecTime]][[3]] + RecurRes[[RecTime]][[2]] / 2,
    (RecurRes[[RecTime]][[3]] * RecurRes[[RecTime]][[1]] - (RecurRes[[RecTime]][[2]] / 2)^2) /
     ((RecurRes[[RecTime]][[3]] + RecurRes[[RecTime]][[2]] / 2)
       (RecurRes[[RecTime]][[1]] + RecurRes[[RecTime]][[2]] / 2))}, {j, 0, 10}];
pA3recσ1 = Partition[Drop[Flatten[Vrin], {3, Length[Flatten[Vrin]], 3}], 2];
F3recσ1 = Partition[Drop[Flatten[Vrin], {2, Length[Flatten[Vrin]] - 1, 3}], 2];

Clear[Vrin];
σ = σit[[2]];
Vrin = Table[Clear[RecurRes, γ];
   γ = γit[[j + 1]];
   RecurRes = NestList[RecurGCA, {1 - freqAa, freqAa, 0}, RecTime] // N;
   {γ, RecurRes[[RecTime]][[3]] + RecurRes[[RecTime]][[2]] / 2,
    (RecurRes[[RecTime]][[3]] * RecurRes[[RecTime]][[1]] - (RecurRes[[RecTime]][[2]] / 2)^2) /
     ((RecurRes[[RecTime]][[3]] + RecurRes[[RecTime]][[2]] / 2)
       (RecurRes[[RecTime]][[1]] + RecurRes[[RecTime]][[2]] / 2))}, {j, 0, 10}];
pA3recσ05 = Partition[Drop[Flatten[Vrin], {3, Length[Flatten[Vrin]], 3}], 2];
F3recσ05 = Partition[Drop[Flatten[Vrin], {2, Length[Flatten[Vrin]] - 1, 3}], 2];

Clear[Vrin];
σ = σit[[3]];
Vrin = Table[Clear[RecurRes, γ];
   γ = γit[[j + 1]];
   RecurRes = NestList[RecurGCA, {1 - freqAa, freqAa, 0}, RecTime] // N;
   {γ, RecurRes[[RecTime]][[3]] + RecurRes[[RecTime]][[2]] / 2,
    (RecurRes[[RecTime]][[3]] * RecurRes[[RecTime]][[1]] - (RecurRes[[RecTime]][[2]] / 2)^2) /
     ((RecurRes[[RecTime]][[3]] + RecurRes[[RecTime]][[2]] / 2)
       (RecurRes[[RecTime]][[1]] + RecurRes[[RecTime]][[2]] / 2))}, {j, 0, 10}];
pA3recσ001 = Partition[Drop[Flatten[Vrin], {3, Length[Flatten[Vrin]], 3}], 2];
F3recσ001 = Partition[Drop[Flatten[Vrin], {2, Length[Flatten[Vrin]] - 1, 3}], 2];
```

In[ ]:=
```
p5σ1 = ListPlot[pA3recσ1, PlotStyle → {Red, PointSize[Large]}];
p5σ05 = ListPlot[pA3recσ05, PlotStyle → {Orange, PointSize[Large]}];
p5σ001 = ListPlot[pA3recσ001, PlotStyle → {Blue, PointSize[Large]}];

p6σ1 = Plot[{pAnumAsex[μ, s, h, 1, γin]},
   {γin, 0, 0.1}, PlotRange → Full, PlotStyle → {{Red}}];
p6σ05 = Plot[{pAnumAsex[μ, s, h, 0.5, γin]},
   {γin, 0, 0.1}, PlotRange → Full, PlotStyle → {{Orange}}];
p6σ001 = Plot[{pAnumAsex[μ, s, h, 0.01, γin]},
   {γin, 0, 0.1}, PlotRange → Full, PlotStyle → {{Blue}}];
```

In[ ]:=
```
plotpA3 = Labeled[Show[p6σ001, p5σ001, p5σ05, p6σ05, p5σ1, p6σ1, ImageSize → 550,
    LabelStyle → {FontFamily → "Arial", FontSize → 18, FontColor → Black}],
   {Text@TraditionalForm@Style["Frequency\nOf Deleterious\nAllele", 18], Text@
     TraditionalForm@Style["Mitotic Gene Conversion, γ", 18]}, {Left, Bottom}]
```

Out[ ]=

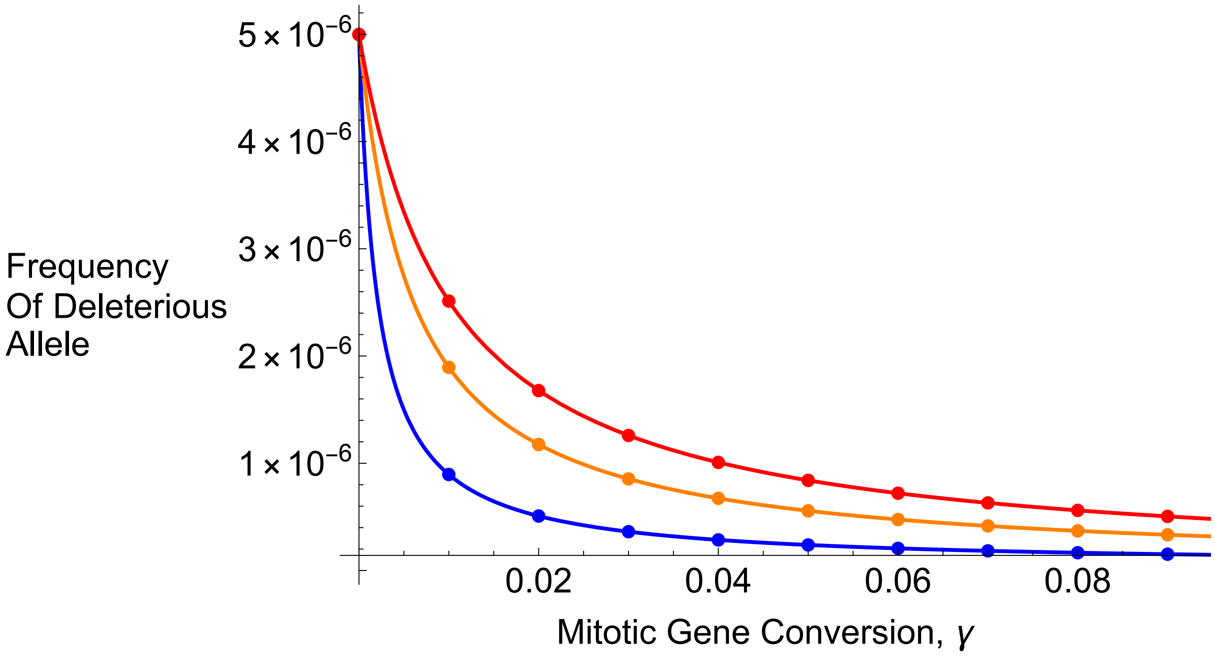

In[ ]:=
```
p5Fσ1 = ListPlot[F3recσ1, PlotStyle → {Red, PointSize[Large]}];
p5Fσ05 = ListPlot[F3recσ05, PlotStyle → {Orange, PointSize[Large]}];
p5Fσ001 = ListPlot[F3recσ001, PlotStyle → {Blue, PointSize[Large]}];

p6Fσ1 = Plot[{FnumAsex[s, 1, γin]},
   {γin, 0, 0.1}, PlotRange → Full, PlotStyle → {{Red}}];
p6Fσ05 = Plot[{FnumAsex[s, 0.5, γin]},
   {γin, 0, 0.1}, PlotRange → Full, PlotStyle → {{Orange}}];
p6Fσ001 = Plot[{FnumAsex[s, 0.01, γin]},
   {γin, 0, 0.1}, PlotRange → Full, PlotStyle → {{Blue}}];
```

In[∘]:=
```
plotF3 =
  Labeled[Show[p6Fσ001, p5Fσ001, p5Fσ1, p6Fσ1, p5Fσ05, p6Fσ05, ImageSize → 550,
    LabelStyle → {FontFamily → "Arial", FontSize → 18, FontColor → Black}],
   {Text@TraditionalForm@Style["Inbreeding\nCoefficient", 18], Text@
     TraditionalForm@Style["Mitotic Gene Conversion, γ", 18]}, {Left, Bottom}]
```

Out[∘]=

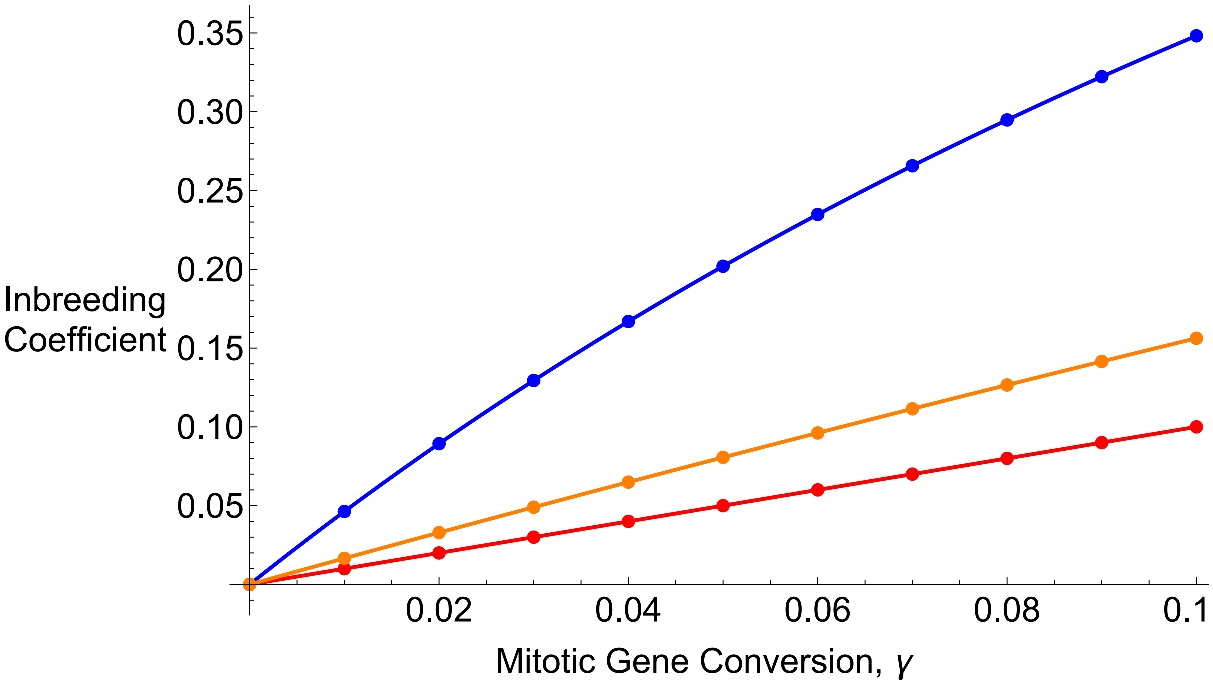


```
Export["RecPlotpA_h001_s02.pdf", plotpA3];
Export["RecPlotpA_h001_s02.jpg", plotpA3];

Export["RecPlotF_h001_s02.pdf", plotF3];
Export["RecPlotF_h001_s02.jpg", plotF3];
```

h = 0.5, s = 0.2 plots

In[∘]:=
```
Clear[freqAa, μ, s, h, RecTime]
freqAa = 0.001;
μ = 10^-8;
s = 0.2;
h = 0.5;
RecTime = 50 000;
```

```
In[•]:= Clear[Vrin];
σ = σit〚1〛;
Vrin = Table[Clear[RecurRes, γ];
   γ = γit〚j + 1〛;
   RecurRes = NestList[RecurGCA, {1 - freqAa, freqAa, 0}, RecTime] // N;
   {γ, RecurRes〚RecTime〛〚3〛 + RecurRes〚RecTime〛〚2〛 / 2,
    (RecurRes〚RecTime〛〚3〛 * RecurRes〚RecTime〛〚1〛 - (RecurRes〚RecTime〛〚2〛 / 2)^2) /
     ((RecurRes〚RecTime〛〚3〛 + RecurRes〚RecTime〛〚2〛 / 2)
       (RecurRes〚RecTime〛〚1〛 + RecurRes〚RecTime〛〚2〛 / 2))}, {j, 0, 10}];
pA4recσ1 = Partition[Drop[Flatten[Vrin], {3, Length[Flatten[Vrin]], 3}], 2];
F4recσ1 = Partition[Drop[Flatten[Vrin], {2, Length[Flatten[Vrin]] - 1, 3}], 2];

Clear[Vrin];
σ = σit〚2〛;
Vrin = Table[Clear[RecurRes, γ];
   γ = γit〚j + 1〛;
   RecurRes = NestList[RecurGCA, {1 - freqAa, freqAa, 0}, RecTime] // N;
   {γ, RecurRes〚RecTime〛〚3〛 + RecurRes〚RecTime〛〚2〛 / 2,
    (RecurRes〚RecTime〛〚3〛 * RecurRes〚RecTime〛〚1〛 - (RecurRes〚RecTime〛〚2〛 / 2)^2) /
     ((RecurRes〚RecTime〛〚3〛 + RecurRes〚RecTime〛〚2〛 / 2)
       (RecurRes〚RecTime〛〚1〛 + RecurRes〚RecTime〛〚2〛 / 2))}, {j, 0, 10}];
pA4recσ05 = Partition[Drop[Flatten[Vrin], {3, Length[Flatten[Vrin]], 3}], 2];
F4recσ05 = Partition[Drop[Flatten[Vrin], {2, Length[Flatten[Vrin]] - 1, 3}], 2];

Clear[Vrin];
σ = σit〚3〛;
Vrin = Table[Clear[RecurRes, γ];
   γ = γit〚j + 1〛;
   RecurRes = NestList[RecurGCA, {1 - freqAa, freqAa, 0}, RecTime] // N;
   {γ, RecurRes〚RecTime〛〚3〛 + RecurRes〚RecTime〛〚2〛 / 2,
    (RecurRes〚RecTime〛〚3〛 * RecurRes〚RecTime〛〚1〛 - (RecurRes〚RecTime〛〚2〛 / 2)^2) /
     ((RecurRes〚RecTime〛〚3〛 + RecurRes〚RecTime〛〚2〛 / 2)
       (RecurRes〚RecTime〛〚1〛 + RecurRes〚RecTime〛〚2〛 / 2))}, {j, 0, 10}];
pA4recσ001 = Partition[Drop[Flatten[Vrin], {3, Length[Flatten[Vrin]], 3}], 2];
F4recσ001 = Partition[Drop[Flatten[Vrin], {2, Length[Flatten[Vrin]] - 1, 3}], 2];
```

```
In[•]:= p7σ1 = ListPlot[pA4recσ1, PlotStyle → {Red, PointSize[Large]}];
p7σ05 = ListPlot[pA4recσ05, PlotStyle → {Orange, PointSize[Large]}];
p7σ001 = ListPlot[pA4recσ001, PlotStyle → {Blue, PointSize[Large]}];

p8σ1 = Plot[{pAnumAsex[μ, s, h, 1, γin]},
   {γin, 0, 0.1}, PlotRange → Full, PlotStyle → {{Red}}];
p8σ05 = Plot[{pAnumAsex[μ, s, h, 0.5, γin]},
   {γin, 0, 0.1}, PlotRange → Full, PlotStyle → {{Orange}}];
p8σ001 = Plot[{pAnumAsex[μ, s, h, 0.01, γin]},
   {γin, 0, 0.1}, PlotRange → Full, PlotStyle → {{Blue}}];
```

In[•]:=
```
plotpA4 = Labeled[Show[p8σ001, p7σ001, p7σ05, p8σ05, p7σ1, p8σ1, ImageSize → 550,
    LabelStyle → {FontFamily → "Arial", FontSize → 18, FontColor → Black}],
   {Text@TraditionalForm@Style["Frequency\nOf Deleterious\nAllele", 18], Text@
     TraditionalForm@Style["Mitotic Gene Conversion, γ", 18]}, {Left, Bottom}]
```

Out[•]=

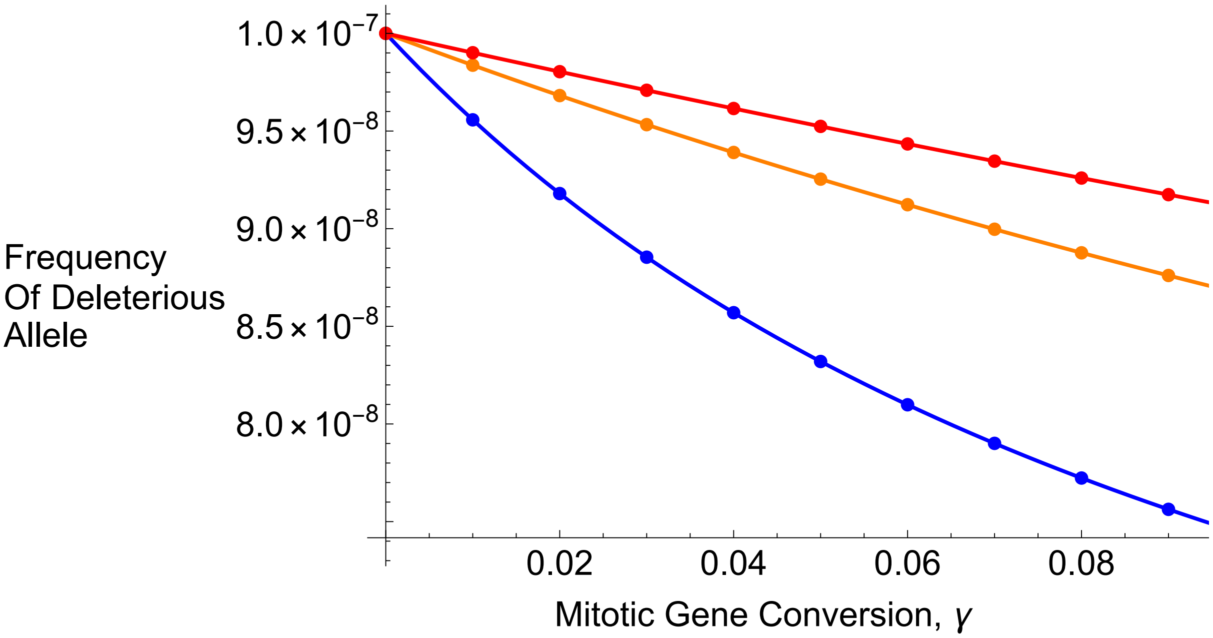

In[•]:=
```
p7Fσ1 = ListPlot[F4recσ1, PlotStyle → {Red, PointSize[Large]}];
p7Fσ05 = ListPlot[F4recσ05, PlotStyle → {Orange, PointSize[Large]}];
p7Fσ001 = ListPlot[F4recσ001, PlotStyle → {Blue, PointSize[Large]}];

p8Fσ1 = Plot[{FnumAsex[s, 1, γin]},
   {γin, 0, 0.1}, PlotRange → Full, PlotStyle → {{Red}}];
p8Fσ05 = Plot[{FnumAsex[s, 0.5, γin]},
   {γin, 0, 0.1}, PlotRange → Full, PlotStyle → {{Orange}}];
p8Fσ001 = Plot[{FnumAsex[s, 0.01, γin]},
   {γin, 0, 0.1}, PlotRange → Full, PlotStyle → {{Blue}}];
```

In[ ]:=
```
plotF4 =
  Labeled[Show[p7Fσ001, p8Fσ001, p7Fσ1, p8Fσ1, p7Fσ05, p8Fσ05, ImageSize → 550,
    LabelStyle → {FontFamily → "Arial", FontSize → 18, FontColor → Black}],
   {Text@TraditionalForm@Style["Inbreeding\nCoefficient", 18], Text@
     TraditionalForm@Style["Mitotic Gene Conversion, γ", 18]}, {Left, Bottom}]
```

Out[ ]=

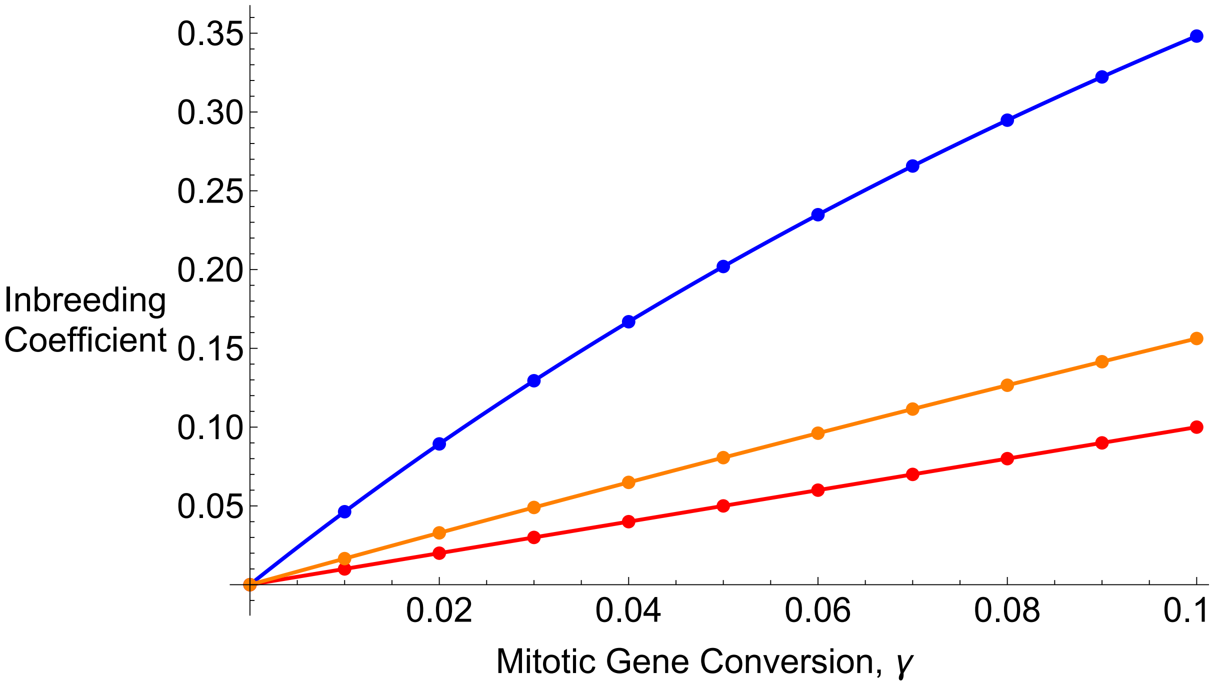


```
Export["RecPlotpA_h05_s02.pdf", plotpA4];
Export["RecPlotpA_h05_s02.jpg", plotpA4];

Export["RecPlotF_h05_s02.pdf", plotF4];
Export["RecPlotF_h05_s02.jpg", plotF4];
```

Numerical recursions, self-fertilisation

Below is the function for a change in genotype frequencies over a single generation:

```
In[ ]:= RecurGCS[{gaa_, gAa_, gAA_}] :=
  {-(((-1 + μ) (4 gaa^2 (1 + (-1 + γ) μ) + gAa (-1 + h s) (gAA (-1 + s)
              (2 γ + S (-1 + γ) (-1 + μ)) + gAa (-1 + h s) (1 + γ - μ + γ μ)) +
          gaa (-4 gAA (-1 + s) (γ + S (-1 + γ) (-1 + μ)) - gAa (-1 + h s)
              (S (-1 + γ) (-1 + μ) + 2 (2 + γ - 2 μ + 2 γ μ))))) /
      (4 (gaa + gAa + gAA - gAA s - gAa h s)^2)), ((-1 + γ) (-1 + μ)
      (4 gaa^2 μ + gAa (-1 + h s) (gAA (-1 + s) (2 + S (-1 + μ)) + gAa (-1 + h s) (1 + μ)) +
        gaa (-4 gAA (-1 + s) (1 + S (-1 + μ)) - gAa (-1 + h s) (2 + S (-1 + μ) + 4 μ)))) /
    (2 (gaa + gAa + gAA - gAA s - gAa h s)^2),
   -((-4 gAA^2 (-1 + s)^2 - 4 gaa gAA (-1 + s) (γ (-1 + μ) + S (-1 + γ) (-1 + μ)^2 - 2 μ) +
        4 gaa^2 (γ (-1 + μ) - μ) μ + gAa^2 (-1 + h s)^2 (-1 + γ (-1 + μ) - μ) (1 + μ) +
        gAa (-1 + h s) (gAA (-1 + s) (2 γ (-1 + μ) + S (-1 + γ) (-1 + μ)^2 - 4 (1 + μ)) +
           gaa (-S (-1 + γ) (-1 + μ)^2 + 4 μ (1 + μ) + 2 γ (1 + μ - 2 μ^2)))) /
      (4 (gaa + gAa + gAA - gAA s - gAa h s)^2))};
```

Defining range of S to be evaluated over:

```
In[ ]:= Sit = {0, 0.5, 0.99};
```

h = 0.01, s = 0.02 plots

```
In[ ]:= Clear[freqAa, μ, s, h, RecTime]
        freqAa = 0.001;
        μ = 10^-8;
        s = 0.02;
        h = 0.01;
        RecTime = 50 000;
```

```
In[ ]:= Clear[Vrin];
S = Sit[[1]];
Vrin = Table[Clear[RecurRes, γ];
   γ = γit[[j + 1]];
   RecurRes = NestList[RecurGCS, {1 - freqAa, freqAa, 0}, RecTime] // N;
   {γ, RecurRes[[RecTime]][[3]] + RecurRes[[RecTime]][[2]] / 2,
    (RecurRes[[RecTime]][[3]] * RecurRes[[RecTime]][[1]] - (RecurRes[[RecTime]][[2]] / 2)^2) /
     ((RecurRes[[RecTime]][[3]] + RecurRes[[RecTime]][[2]] / 2)
       (RecurRes[[RecTime]][[1]] + RecurRes[[RecTime]][[2]] / 2))}, {j, 0, 10}];
pArecS0 = Partition[Drop[Flatten[Vrin], {3, Length[Flatten[Vrin]], 3}], 2];
FrecS0 = Partition[Drop[Flatten[Vrin], {2, Length[Flatten[Vrin]] - 1, 3}], 2];

Clear[Vrin];
S = Sit[[2]];
Vrin = Table[Clear[RecurRes, γ];
   γ = γit[[j + 1]];
   RecurRes = NestList[RecurGCS, {1 - freqAa, freqAa, 0}, RecTime] // N;
   {γ, RecurRes[[RecTime]][[3]] + RecurRes[[RecTime]][[2]] / 2,
    (RecurRes[[RecTime]][[3]] * RecurRes[[RecTime]][[1]] - (RecurRes[[RecTime]][[2]] / 2)^2) /
     ((RecurRes[[RecTime]][[3]] + RecurRes[[RecTime]][[2]] / 2)
       (RecurRes[[RecTime]][[1]] + RecurRes[[RecTime]][[2]] / 2))}, {j, 0, 10}];
pArecS05 = Partition[Drop[Flatten[Vrin], {3, Length[Flatten[Vrin]], 3}], 2];
FrecS05 = Partition[Drop[Flatten[Vrin], {2, Length[Flatten[Vrin]] - 1, 3}], 2];

Clear[Vrin];
S = Sit[[3]];
Vrin = Table[Clear[RecurRes, γ];
   γ = γit[[j + 1]];
   RecurRes = NestList[RecurGCS, {1 - freqAa, freqAa, 0}, RecTime] // N;
   {γ, RecurRes[[RecTime]][[3]] + RecurRes[[RecTime]][[2]] / 2,
    (RecurRes[[RecTime]][[3]] * RecurRes[[RecTime]][[1]] - (RecurRes[[RecTime]][[2]] / 2)^2) /
     ((RecurRes[[RecTime]][[3]] + RecurRes[[RecTime]][[2]] / 2)
       (RecurRes[[RecTime]][[1]] + RecurRes[[RecTime]][[2]] / 2))}, {j, 0, 10}];
pArecS099 = Partition[Drop[Flatten[Vrin], {3, Length[Flatten[Vrin]], 3}], 2];
FrecS099 = Partition[Drop[Flatten[Vrin], {2, Length[Flatten[Vrin]] - 1, 3}], 2];
```

In[ ]:=
```
p1S0 = ListPlot[pArecS0, PlotStyle → {Red, PointSize[Large]}, PlotLegends →
    Placed[PointLegend[{Directive[Red, AbsolutePointSize[8]], Directive[Orange,
        AbsolutePointSize[8]], Directive[Blue, AbsolutePointSize[8]]},
      {"S = 0", "S = 0.5", "S = 0.99"}], {0.4, 0.7}], LabelStyle → 18];
p1S05 = ListPlot[pArecS05, PlotStyle → {Orange, PointSize[Large]},
   PlotLegends → Placed[PointLegend[{Directive[Black, AbsolutePointSize[8]]},
      {"Numerical Recursions"}], {0.8, 0.8}], LabelStyle → 18];
p1S099 = ListPlot[pArecS099, PlotStyle → {Blue, PointSize[Large]}];

p2S0 = Plot[{pAnumSelf[μ, s, h, 0, γin]},
   {γin, 0, 0.1}, PlotRange → Full, PlotStyle → {{Red}}, PlotLegends →
    Placed[LineLegend[{Black}, {"Equation 1"}, LabelStyle → 18], {0.75, 0.65}]];
p2S05 = Plot[{pAnumSelf[μ, s, h, 0.5, γin]},
   {γin, 0, 0.1}, PlotRange → Full, PlotStyle → {{Orange}}];
p2S099 = Plot[{pAnumSelf[μ, s, h, 0.99, γin]},
   {γin, 0, 0.1}, PlotRange → Full, PlotStyle → {{Blue}}];
```

In[ ]:=
```
plotpS1 = Labeled[Show[p1S0, p2S0, p1S05, p2S05, p1S099, p2S099, ImageSize → 550,
   LabelStyle → {FontFamily → "Arial", FontSize → 18, FontColor → Black}],
  {Text@TraditionalForm@Style["Frequency\nOf Deleterious\nAllele", 18], Text@
    TraditionalForm@Style["Mitotic Gene Conversion, γ", 18]}, {Left, Bottom}]
```

Out[ ]=

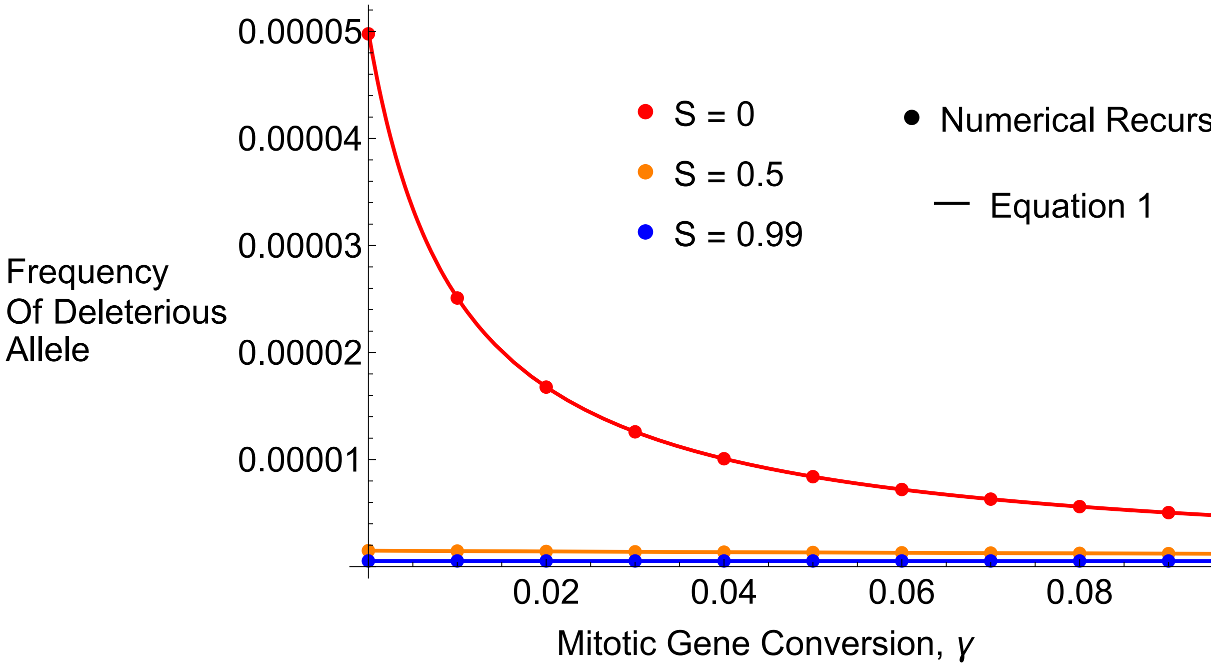

In[ ]:=
```
Export["Figures_Jan2025/RecursionsSelf/RecPlotpA_h001_s002_Self.pdf", plotpS1];
Export["Figures_Jan2025/RecursionsSelf/RecPlotpA_h001_s002_Self.jpg", plotpS1];
```

```
In[•]:= p1FS0 = ListPlot[FrecS0, PlotStyle → {Red, PointSize[Large]}];
        p1FS05 = ListPlot[FrecS05, PlotStyle → {Orange, PointSize[Large]}];
        p1FS099 = ListPlot[FrecS099, PlotStyle → {Blue, PointSize[Large]}];

        p2FS0 = Plot[{FnumSelf[s, 0, γin, 0.01]}, {γin, 0, 0.1},
           PlotRange → {Full, {-0.05, 1}}, PlotStyle → {{Red}}, PlotLegends →
            Placed[LineLegend[{Black}, {"Equation 3"}, LabelStyle → 18], {0.75, 0.65}]];
        p2FS05 = Plot[{FnumSelf[s, 0.5, γin, 0.01]}, {γin, 0, 0.1},
           PlotRange → {Full, {-0.05, 1}}, PlotStyle → {{Orange}}];
        p2FS099 = Plot[{FnumSelf[s, 0.99, γin, 0.01]},
           {γin, 0, 0.1}, PlotRange → {Full, {-0.05, 1}}, PlotStyle → {{Blue}}];
```

```
In[•]:= plotF1S =
         Labeled[Show[p2FS099, p1FS099, p1FS0, p2FS0, p1FS05, p2FS05, ImageSize → 550,
           LabelStyle → {FontFamily → "Arial", FontSize → 18, FontColor → Black}],
          {Text@TraditionalForm@Style["Inbreeding\nCoefficient", 18], Text@
            TraditionalForm@Style["Mitotic Gene Conversion, γ", 18]}, {Left, Bottom}]
```

Out[•]=

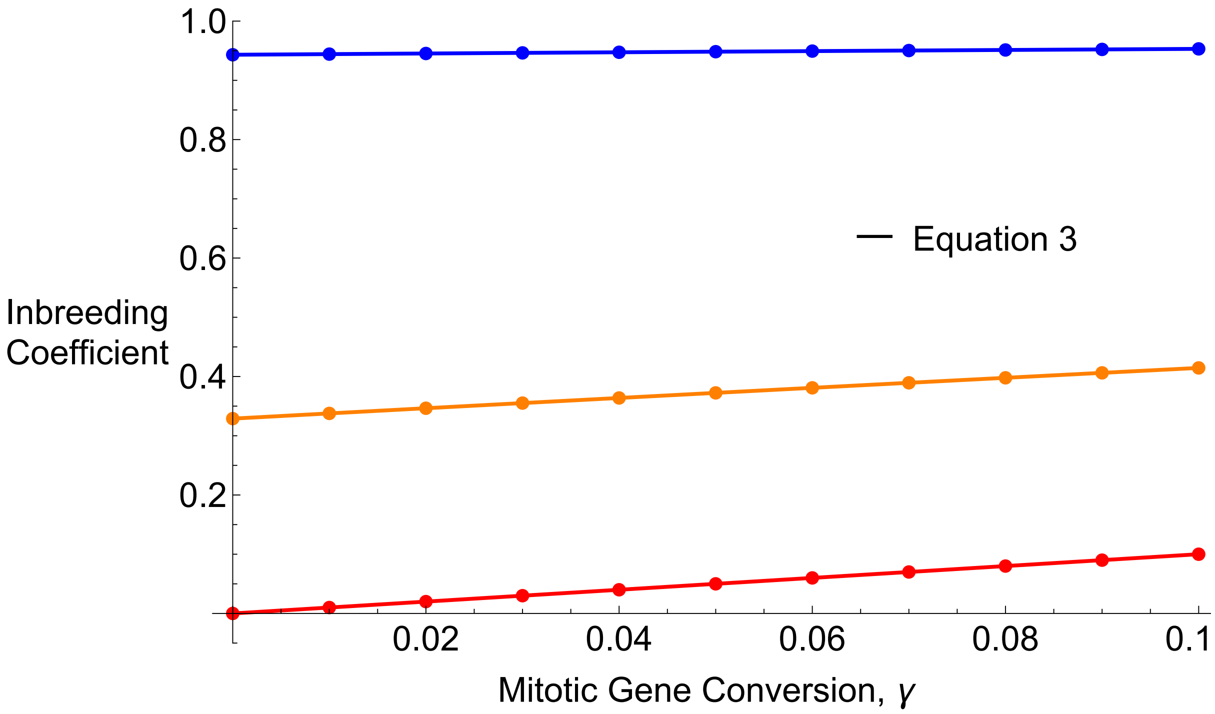


```
In[•]:= Export["Figures_Jan2025/RecursionsSelf/RecPlotF_h001_s002_Self.pdf", plotF1S];
        Export["Figures_Jan2025/RecursionsSelf/RecPlotF_h001_s002_Self.jpg", plotF1S];
```

h = 0.5, s = 0.02 plots

```
In[•]:= Clear[freqAa, μ, s, h, RecTime]
        freqAa = 0.001;
        μ = 10^-8;
        s = 0.02;
        h = 0.5;
        RecTime = 50 000;
```

```
In[ ]:= Clear[Vrin];
S = Sit[[1]];
Vrin = Table[Clear[RecurRes, γ];
   γ = γit[[j + 1]];
   RecurRes = NestList[RecurGCS, {1 - freqAa, freqAa, 0}, RecTime] // N;
   {γ, RecurRes[[RecTime]][[3]] + RecurRes[[RecTime]][[2]] / 2,
    (RecurRes[[RecTime]][[3]] * RecurRes[[RecTime]][[1]] - (RecurRes[[RecTime]][[2]] / 2)^2) /
     ((RecurRes[[RecTime]][[3]] + RecurRes[[RecTime]][[2]] / 2)
       (RecurRes[[RecTime]][[1]] + RecurRes[[RecTime]][[2]] / 2))}, {j, 0, 10}];
pA2recS0 = Partition[Drop[Flatten[Vrin], {3, Length[Flatten[Vrin]], 3}], 2];
F2recS0 = Partition[Drop[Flatten[Vrin], {2, Length[Flatten[Vrin]] - 1, 3}], 2];

Clear[Vrin];
S = Sit[[2]];
Vrin = Table[Clear[RecurRes, γ];
   γ = γit[[j + 1]];
   RecurRes = NestList[RecurGCS, {1 - freqAa, freqAa, 0}, RecTime] // N;
   {γ, RecurRes[[RecTime]][[3]] + RecurRes[[RecTime]][[2]] / 2,
    (RecurRes[[RecTime]][[3]] * RecurRes[[RecTime]][[1]] - (RecurRes[[RecTime]][[2]] / 2)^2) /
     ((RecurRes[[RecTime]][[3]] + RecurRes[[RecTime]][[2]] / 2)
       (RecurRes[[RecTime]][[1]] + RecurRes[[RecTime]][[2]] / 2))}, {j, 0, 10}];
pA2recS05 = Partition[Drop[Flatten[Vrin], {3, Length[Flatten[Vrin]], 3}], 2];
F2recS05 = Partition[Drop[Flatten[Vrin], {2, Length[Flatten[Vrin]] - 1, 3}], 2];

Clear[Vrin];
S = Sit[[3]];
Vrin = Table[Clear[RecurRes, γ];
   γ = γit[[j + 1]];
   RecurRes = NestList[RecurGCS, {1 - freqAa, freqAa, 0}, RecTime] // N;
   {γ, RecurRes[[RecTime]][[3]] + RecurRes[[RecTime]][[2]] / 2,
    (RecurRes[[RecTime]][[3]] * RecurRes[[RecTime]][[1]] - (RecurRes[[RecTime]][[2]] / 2)^2) /
     ((RecurRes[[RecTime]][[3]] + RecurRes[[RecTime]][[2]] / 2)
       (RecurRes[[RecTime]][[1]] + RecurRes[[RecTime]][[2]] / 2))}, {j, 0, 10}];
pA2recS099 = Partition[Drop[Flatten[Vrin], {3, Length[Flatten[Vrin]], 3}], 2];
F2recS099 = Partition[Drop[Flatten[Vrin], {2, Length[Flatten[Vrin]] - 1, 3}], 2];
```

In[ ]:=
```
p3S0 = ListPlot[pA2recS0, PlotStyle → {Red, PointSize[Large]}];
p3S05 = ListPlot[pA2recS05, PlotStyle → {Orange, PointSize[Large]}];
p3S099 = ListPlot[pA2recS099, PlotStyle → {Blue, PointSize[Large]}];

p4S0 = Plot[{pAnumSelf[μ, s, h, 0, γin]}, {γin, 0, 0.1},
   PlotRange → {Full, {0, μ/(h s) (1.05)}}, PlotStyle → {{Red}}];
p4S05 = Plot[{pAnumSelf[μ, s, h, 0.5, γin]}, {γin, 0, 0.1},
   PlotRange → {Full, {0, μ/(h s) (1.05)}}, PlotStyle → {{Orange}}];
p4S099 = Plot[{pAnumSelf[μ, s, h, 0.99, γin]}, {γin, 0, 0.1},
   PlotRange → {Full, {0, μ/(h s) (1.05)}}, PlotStyle → {{Blue}}];
```

In[ ]:=
```
plotpS2 = Labeled[Show[p4S0, p3S0, p3S05, p4S05, p4S099, p3S099, ImageSize → 550,
   LabelStyle → {FontFamily → "Arial", FontSize → 18, FontColor → Black}],
  {Text@TraditionalForm@Style["Frequency\nOf Deleterious\nAllele", 18], Text@
    TraditionalForm@Style["Mitotic Gene Conversion, γ", 18]}, {Left, Bottom}]
```

Out[ ]=

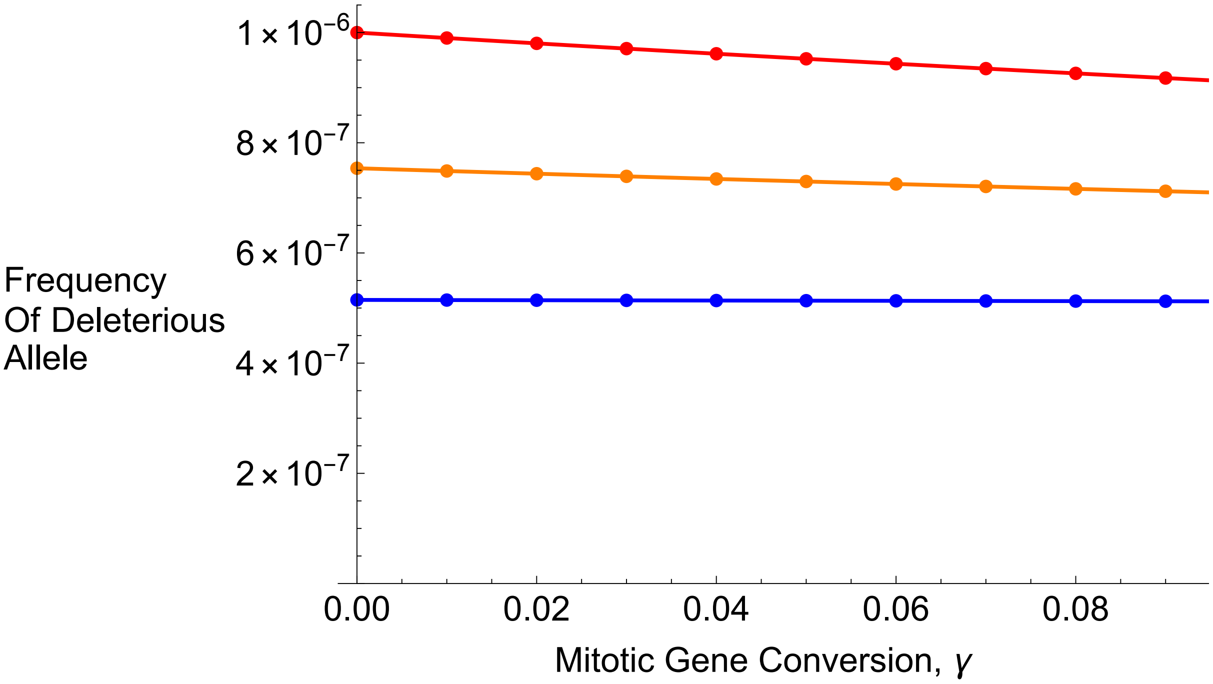

In[ ]:=
```
p3FS0 = ListPlot[F2recS0, PlotStyle → {Red, PointSize[Large]}];
p3FS05 = ListPlot[F2recS05, PlotStyle → {Orange, PointSize[Large]}];
p3FS099 = ListPlot[F2recS099, PlotStyle → {Blue, PointSize[Large]}];

p4FS0 = Plot[{FnumSelf[s, 0, γin, h]}, {γin, 0, 0.1},
   PlotRange → {Full, {-0.05, 1}}, PlotStyle → {{Red}}];
p4FS05 = Plot[{FnumSelf[s, 0.5, γin, h]}, {γin, 0, 0.1},
   PlotRange → {Full, {-0.05, 1}}, PlotStyle → {{Orange}}];
p4FS099 = Plot[{FnumSelf[s, 0.99, γin, h]}, {γin, 0, 0.1},
   PlotRange → {Full, {-0.05, 1}}, PlotStyle → {{Blue}}];
```

In[∘]:=
```
plotF2S =
  Labeled[Show[p4FS099, p3FS099, p3FS0, p4FS0, p3FS05, p4FS05, ImageSize → 550,
    LabelStyle → {FontFamily → "Arial", FontSize → 18, FontColor → Black}],
   {Text@TraditionalForm@Style["Inbreeding\nCoefficient", 18], Text@
     TraditionalForm@Style["Mitotic Gene Conversion, γ", 18]}, {Left, Bottom}]
```

Out[∘]=

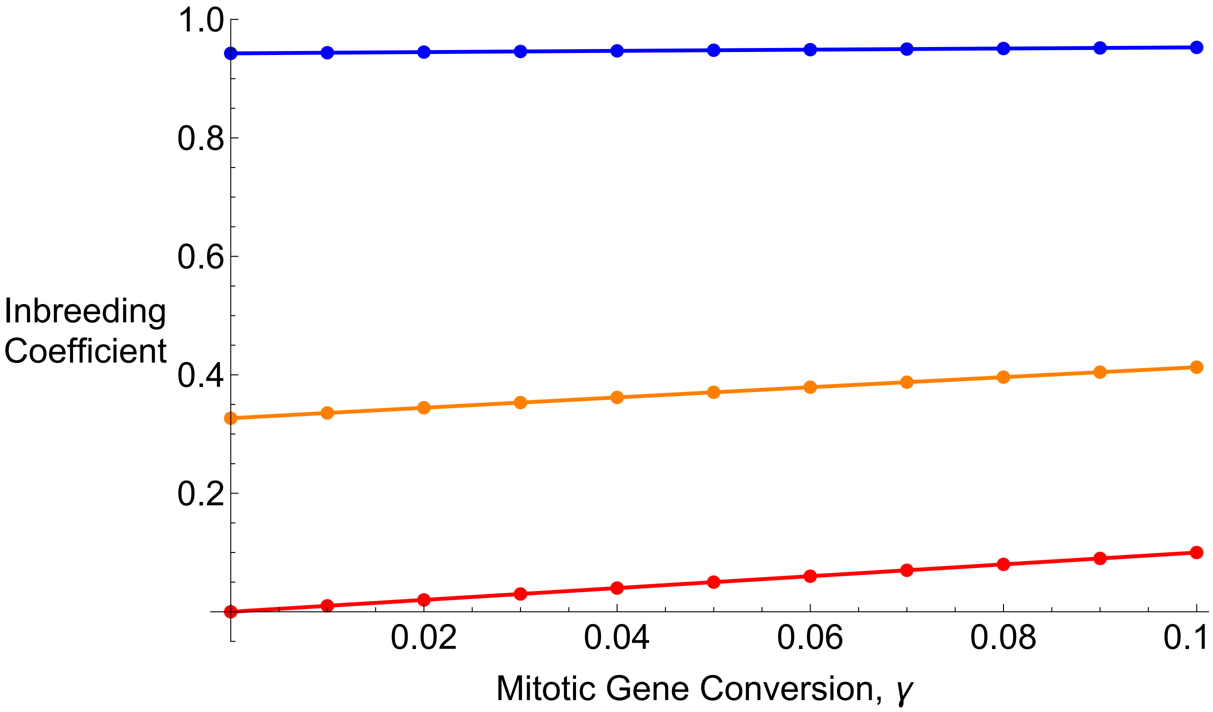

In[∘]:=
```
Export["Figures_Jan2025/RecursionsSelf/RecPlotpA_h05_s002_Self.pdf", plotpS2];
Export["Figures_Jan2025/RecursionsSelf/RecPlotpA_h05_s002_Self.jpg", plotpS2];

Export["Figures_Jan2025/RecursionsSelf/RecPlotF_h05_s002_Self.pdf", plotF2S];
Export["Figures_Jan2025/RecursionsSelf/RecPlotF_h05_s002_Self.jpg", plotF2S];
```

h = 0.01, s = 0.2 plots

In[∘]:=
```
Clear[freqAa, μ, s, h, RecTime]
freqAa = 0.001;
μ = 10^-8;
s = 0.2;
h = 0.01;
RecTime = 50 000;
```

```
In[ ]:= Clear[Vrin];
S = Sit[[1]];
Vrin = Table[Clear[RecurRes, γ];
    γ = γit[[j + 1]];
    RecurRes = NestList[RecurGCS, {1 - freqAa, freqAa, 0}, RecTime] // N;
    {γ, RecurRes[[RecTime]][[3]] + RecurRes[[RecTime]][[2]] / 2,
      (RecurRes[[RecTime]][[3]] * RecurRes[[RecTime]][[1]] - (RecurRes[[RecTime]][[2]] / 2)^2) /
       ((RecurRes[[RecTime]][[3]] + RecurRes[[RecTime]][[2]] / 2)
         (RecurRes[[RecTime]][[1]] + RecurRes[[RecTime]][[2]] / 2))}, {j, 0, 10}];
pA3recS0 = Partition[Drop[Flatten[Vrin], {3, Length[Flatten[Vrin]], 3}], 2];
F3recS0 = Partition[Drop[Flatten[Vrin], {2, Length[Flatten[Vrin]] - 1, 3}], 2];

Clear[Vrin];
S = Sit[[2]];
Vrin = Table[Clear[RecurRes, γ];
    γ = γit[[j + 1]];
    RecurRes = NestList[RecurGCS, {1 - freqAa, freqAa, 0}, RecTime] // N;
    {γ, RecurRes[[RecTime]][[3]] + RecurRes[[RecTime]][[2]] / 2,
      (RecurRes[[RecTime]][[3]] * RecurRes[[RecTime]][[1]] - (RecurRes[[RecTime]][[2]] / 2)^2) /
       ((RecurRes[[RecTime]][[3]] + RecurRes[[RecTime]][[2]] / 2)
         (RecurRes[[RecTime]][[1]] + RecurRes[[RecTime]][[2]] / 2))}, {j, 0, 10}];
pA3recS05 = Partition[Drop[Flatten[Vrin], {3, Length[Flatten[Vrin]], 3}], 2];
F3recS05 = Partition[Drop[Flatten[Vrin], {2, Length[Flatten[Vrin]] - 1, 3}], 2];

Clear[Vrin];
S = Sit[[3]];
Vrin = Table[Clear[RecurRes, γ];
    γ = γit[[j + 1]];
    RecurRes = NestList[RecurGCS, {1 - freqAa, freqAa, 0}, RecTime] // N;
    {γ, RecurRes[[RecTime]][[3]] + RecurRes[[RecTime]][[2]] / 2,
      (RecurRes[[RecTime]][[3]] * RecurRes[[RecTime]][[1]] - (RecurRes[[RecTime]][[2]] / 2)^2) /
       ((RecurRes[[RecTime]][[3]] + RecurRes[[RecTime]][[2]] / 2)
         (RecurRes[[RecTime]][[1]] + RecurRes[[RecTime]][[2]] / 2))}, {j, 0, 10}];
pA3recS099 = Partition[Drop[Flatten[Vrin], {3, Length[Flatten[Vrin]], 3}], 2];
F3recS099 = Partition[Drop[Flatten[Vrin], {2, Length[Flatten[Vrin]] - 1, 3}], 2];
```

In[ ]:=
```
p5S0 = ListPlot[pA3recS0, PlotStyle → {Red, PointSize[Large]}];
p5S05 = ListPlot[pA3recS05, PlotStyle → {Orange, PointSize[Large]}];
p5S099 = ListPlot[pA3recS099, PlotStyle → {Blue, PointSize[Large]}];

p6S0 = Plot[{pAnumSelf[μ, s, h, 0, γin]}, {γin, 0, 0.1},
   PlotRange → {Full, {0, μ/(h s) (1.05)}}, PlotStyle → {{Red}}];
p6S05 = Plot[{pAnumSelf[μ, s, h, 0.5, γin]}, {γin, 0, 0.1},
   PlotRange → {Full, {0, μ/(h s) (1.05)}}, PlotStyle → {{Orange}}];
p6S099 = Plot[{pAnumSelf[μ, s, h, 0.99, γin]}, {γin, 0, 0.1},
   PlotRange → {Full, {0, μ/(h s) (1.05)}}, PlotStyle → {{Blue}}];
```

In[ ]:=
```
plotpS3 = Labeled[Show[p6S099, p5S099, p5S05, p6S05, p5S0, p6S0, ImageSize → 550,
   LabelStyle → {FontFamily → "Arial", FontSize → 18, FontColor → Black}],
  {Text@TraditionalForm@Style["Frequency\nOf Deleterious\nAllele", 18], Text@
    TraditionalForm@Style["Mitotic Gene Conversion, γ", 18]}, {Left, Bottom}]
```

Out[ ]=

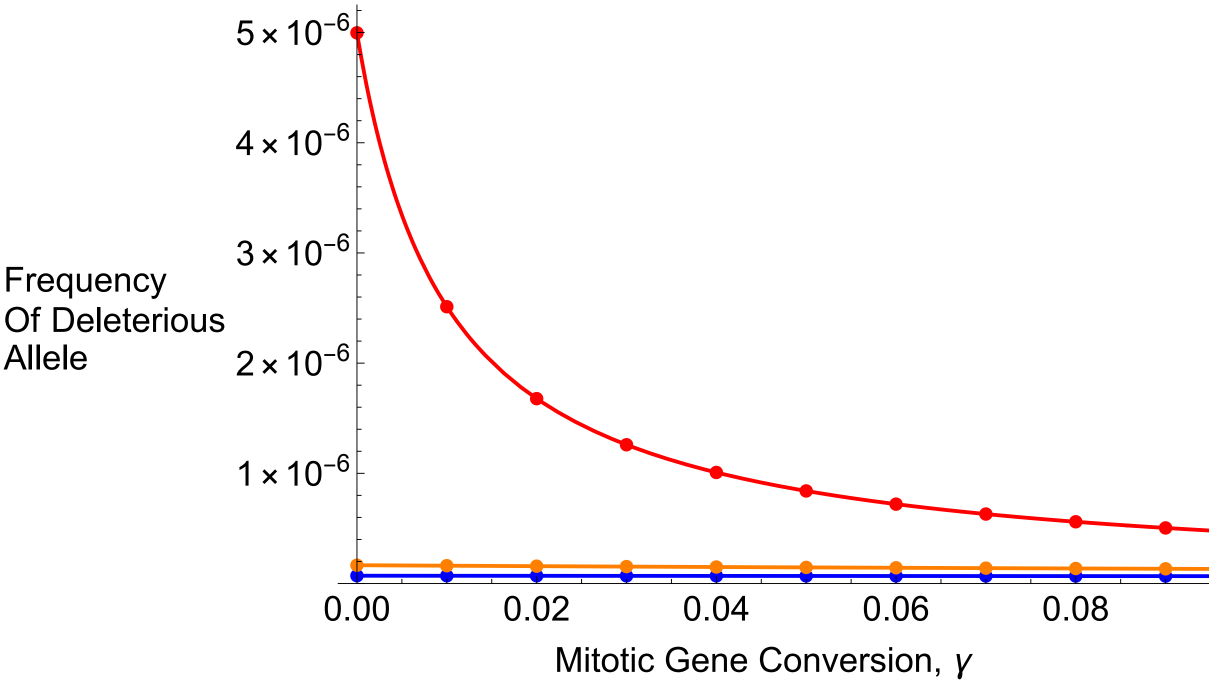

In[ ]:=
```
p5FS0 = ListPlot[F3recS0, PlotStyle → {Red, PointSize[Large]}];
p5FS05 = ListPlot[F3recS05, PlotStyle → {Orange, PointSize[Large]}];
p5FS099 = ListPlot[F3recS099, PlotStyle → {Blue, PointSize[Large]}];

p6FS0 = Plot[{FnumSelf[s, 0, γin, h]}, {γin, 0, 0.1},
   PlotRange → {Full, {-0.05, 1}}, PlotStyle → {{Red}, {Red, Dashed}}];
p6FS05 = Plot[{FnumSelf[s, 0.5, γin, h]}, {γin, 0, 0.1},
   PlotRange → {Full, {-0.05, 1}}, PlotStyle → {{Orange}, {Orange, Dashed}}];
p6FS099 = Plot[{FnumSelf[s, 0.99, γin, h]}, {γin, 0, 0.1},
   PlotRange → {Full, {-0.05, 1}}, PlotStyle → {{Blue}, {Blue, Dashed}}];
```

In[∘]:=
```
plotF3S =
  Labeled[Show[p6FS099, p5FS099, p5FS0, p6FS0, p5FS05, p6FS05, ImageSize → 550,
    LabelStyle → {FontFamily → "Arial", FontSize → 18, FontColor → Black}],
   {Text@TraditionalForm@Style["Inbreeding\nCoefficient", 18], Text@
     TraditionalForm@Style["Mitotic Gene Conversion, γ", 18]}, {Left, Bottom}]
```

Out[∘]=

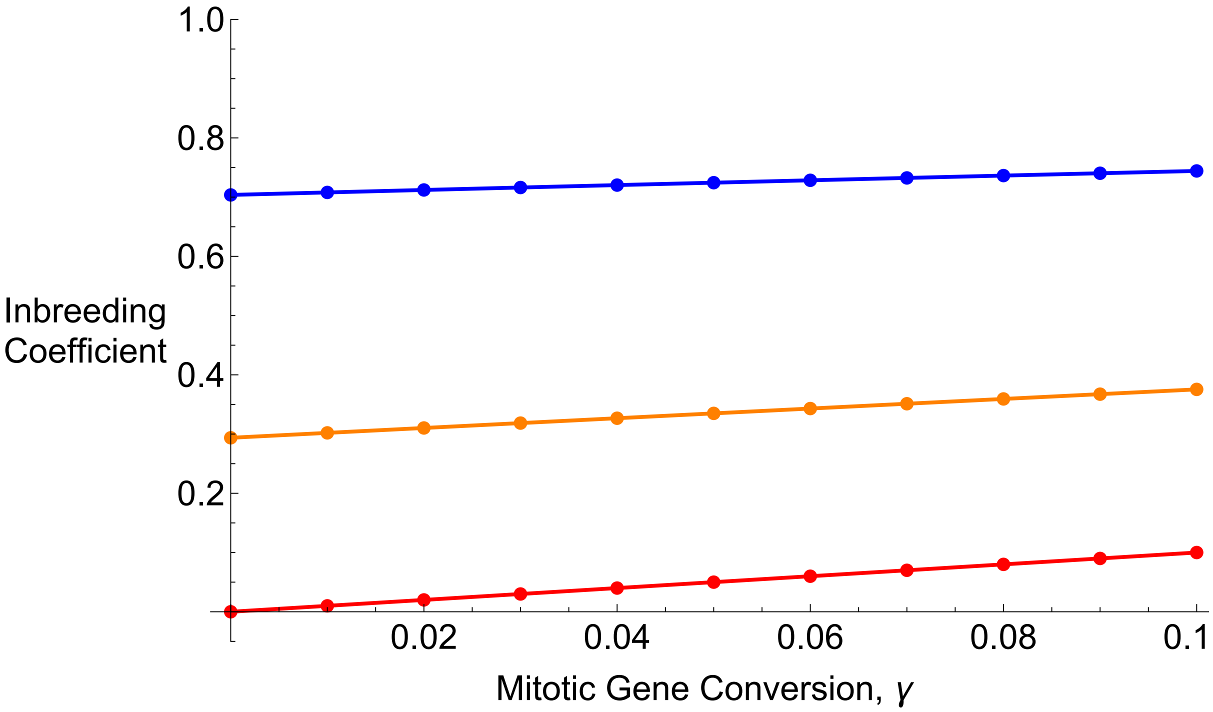

In[∘]:=
```
Export["Figures_Jan2025/RecursionsSelf/RecPlotpA_h001_s02_Self.pdf", plotpS3];
Export["Figures_Jan2025/RecursionsSelf/RecPlotpA_h001_s02_Self.jpg", plotpS3];

Export["Figures_Jan2025/RecursionsSelf/RecPlotF_h001_s02_Self.pdf", plotF3S];
Export["Figures_Jan2025/RecursionsSelf/RecPlotF_h001_s02_Self.jpg", plotF3S];
```

h = 0.5, s = 0.2 plots

In[∘]:=
```
Clear[freqAa, μ, s, h, RecTime]
freqAa = 0.001;
μ = 10^-8;
s = 0.2;
h = 0.5;
RecTime = 50 000;
```

```
In[•]:= Clear[Vrin];
S = Sit[[1]];
Vrin = Table[Clear[RecurRes, γ];
   γ = γit[[j + 1]];
   RecurRes = NestList[RecurGCS, {1 - freqAa, freqAa, 0}, RecTime] // N;
   {γ, RecurRes[[RecTime]][[3]] + RecurRes[[RecTime]][[2]] / 2,
    (RecurRes[[RecTime]][[3]] * RecurRes[[RecTime]][[1]] - (RecurRes[[RecTime]][[2]] / 2)^2) /
     ((RecurRes[[RecTime]][[3]] + RecurRes[[RecTime]][[2]] / 2)
       (RecurRes[[RecTime]][[1]] + RecurRes[[RecTime]][[2]] / 2))}, {j, 0, 10}];
pA4recS0 = Partition[Drop[Flatten[Vrin], {3, Length[Flatten[Vrin]], 3}], 2];
F4recS0 = Partition[Drop[Flatten[Vrin], {2, Length[Flatten[Vrin]] - 1, 3}], 2];

Clear[Vrin];
S = Sit[[2]];
Vrin = Table[Clear[RecurRes, γ];
   γ = γit[[j + 1]];
   RecurRes = NestList[RecurGCS, {1 - freqAa, freqAa, 0}, RecTime] // N;
   {γ, RecurRes[[RecTime]][[3]] + RecurRes[[RecTime]][[2]] / 2,
    (RecurRes[[RecTime]][[3]] * RecurRes[[RecTime]][[1]] - (RecurRes[[RecTime]][[2]] / 2)^2) /
     ((RecurRes[[RecTime]][[3]] + RecurRes[[RecTime]][[2]] / 2)
       (RecurRes[[RecTime]][[1]] + RecurRes[[RecTime]][[2]] / 2))}, {j, 0, 10}];
pA4recS05 = Partition[Drop[Flatten[Vrin], {3, Length[Flatten[Vrin]], 3}], 2];
F4recS05 = Partition[Drop[Flatten[Vrin], {2, Length[Flatten[Vrin]] - 1, 3}], 2];

Clear[Vrin];
S = Sit[[3]];
Vrin = Table[Clear[RecurRes, γ];
   γ = γit[[j + 1]];
   RecurRes = NestList[RecurGCS, {1 - freqAa, freqAa, 0}, RecTime] // N;
   {γ, RecurRes[[RecTime]][[3]] + RecurRes[[RecTime]][[2]] / 2,
    (RecurRes[[RecTime]][[3]] * RecurRes[[RecTime]][[1]] - (RecurRes[[RecTime]][[2]] / 2)^2) /
     ((RecurRes[[RecTime]][[3]] + RecurRes[[RecTime]][[2]] / 2)
       (RecurRes[[RecTime]][[1]] + RecurRes[[RecTime]][[2]] / 2))}, {j, 0, 10}];
pA4recS099 = Partition[Drop[Flatten[Vrin], {3, Length[Flatten[Vrin]], 3}], 2];
F4recS099 = Partition[Drop[Flatten[Vrin], {2, Length[Flatten[Vrin]] - 1, 3}], 2];
```

In[ ]:=
```
p7S0 = ListPlot[pA4recS0, PlotStyle → {Red, PointSize[Large]}];
p7S05 = ListPlot[pA4recS05, PlotStyle → {Orange, PointSize[Large]}];
p7S099 = ListPlot[pA4recS099, PlotStyle → {Blue, PointSize[Large]}];

p8S0 = Plot[{pAnumSelf[μ, s, h, 0, γin]}, {γin, 0, 0.1},
   PlotRange → {Full, {0, μ/(h s) (1.05)}}, PlotStyle → {{Red}, {Red, Dashed}}];
p8S05 = Plot[{pAnumSelf[μ, s, h, 0.5, γin]}, {γin, 0, 0.1},
   PlotRange → {Full, {0, μ/(h s) (1.05)}}, PlotStyle → {{Orange}, {Orange, Dashed}}];
p8S099 = Plot[{pAnumSelf[μ, s, h, 0.99, γin]}, {γin, 0, 0.1},
   PlotRange → {Full, {0, μ/(h s) (1.05)}}, PlotStyle → {{Blue}, {Blue, Dashed}}];
```

In[ ]:=
```
plotpS4 = Labeled[Show[p8S099, p7S099, p7S05, p8S05, p7S0, p8S0, ImageSize → 550,
   LabelStyle → {FontFamily → "Arial", FontSize → 18, FontColor → Black}],
  {Text@TraditionalForm@Style["Frequency\nOf Deleterious\nAllele", 18], Text@
    TraditionalForm@Style["Mitotic Gene Conversion, γ", 18]}, {Left, Bottom}]
```

Out[ ]=

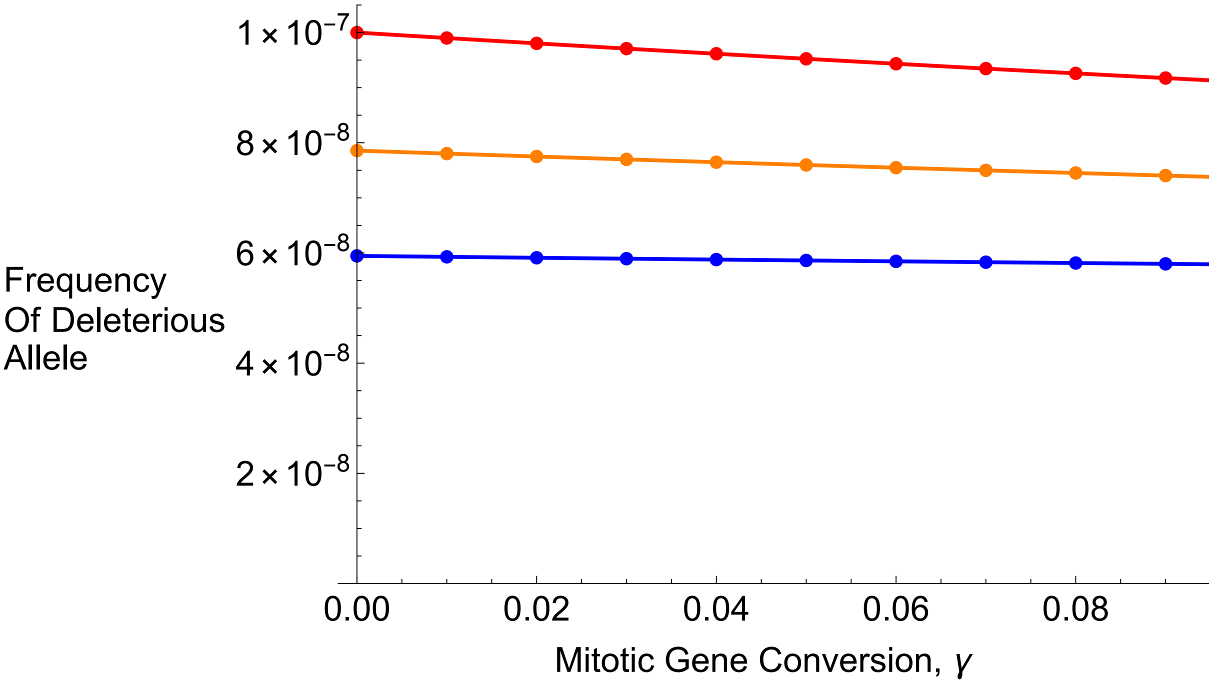

In[ ]:=
```
p7FS0 = ListPlot[F4recS0, PlotStyle → {Red, PointSize[Large]}];
p7FS05 = ListPlot[F4recS05, PlotStyle → {Orange, PointSize[Large]}];
p7FS099 = ListPlot[F4recS099, PlotStyle → {Blue, PointSize[Large]}];

p8FS0 = Plot[{FnumSelf[s, 0, γin, h]}, {γin, 0, 0.1},
   PlotRange → {Full, {-0.05, 1}}, PlotStyle → {{Red}, {Red, Dashed}}];
p8FS05 = Plot[{FnumSelf[s, 0.5, γin, h]}, {γin, 0, 0.1},
   PlotRange → {Full, {-0.05, 1}}, PlotStyle → {{Orange}, {Orange, Dashed}}];
p8FS099 = Plot[{FnumSelf[s, 0.99, γin, h]}, {γin, 0, 0.1},
   PlotRange → {Full, {-0.05, 1}}, PlotStyle → {{Blue}, {Blue, Dashed}}];
```

In[•]:=
```
plotF4S =
  Labeled[Show[p8FS099, p7FS099, p7FS0, p8FS0, p7FS05, p8FS05, ImageSize → 550,
    LabelStyle → {FontFamily → "Arial", FontSize → 18, FontColor → Black}],
   {Text@TraditionalForm@Style["Inbreeding\nCoefficient", 18], Text@
     TraditionalForm@Style["Mitotic Gene Conversion, γ", 18]}, {Left, Bottom}]
```

Out[•]=

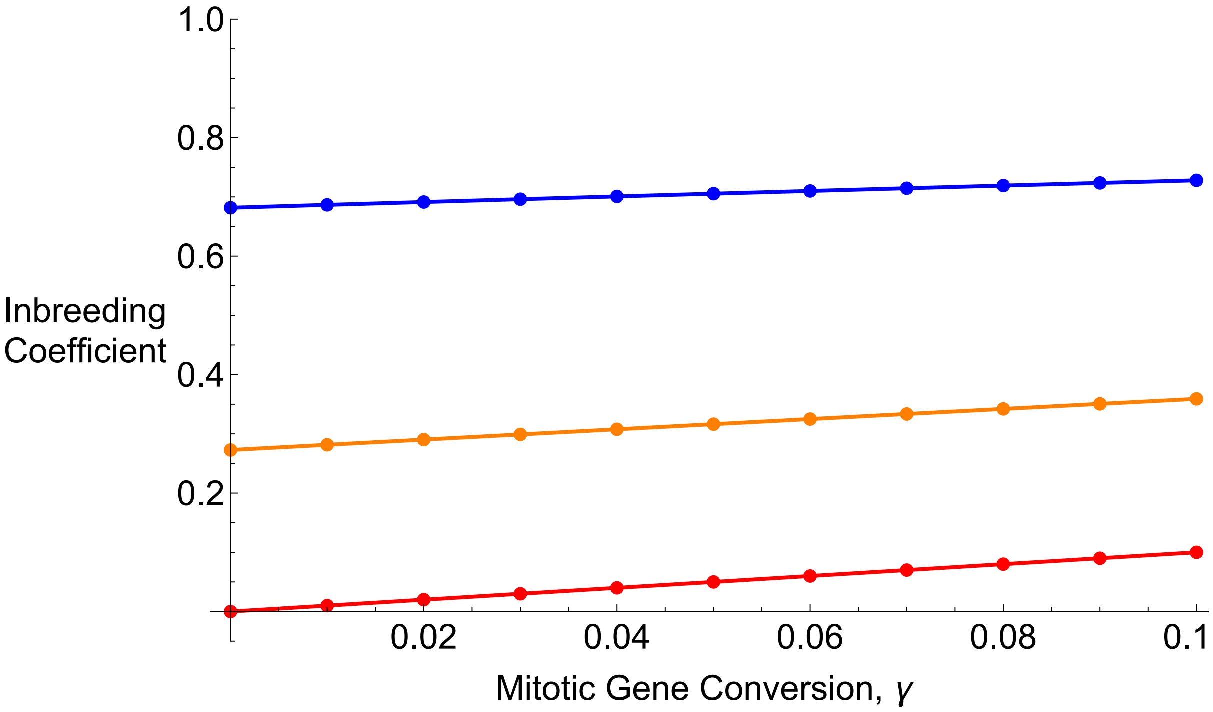

In[•]:=
```
Export["Figures_Jan2025/RecursionsSelf/RecPlotpA_h05_s02_Self.pdf", plotpS4];
Export["Figures_Jan2025/RecursionsSelf/RecPlotpA_h05_s02_Self.jpg", plotpS4];

Export["Figures_Jan2025/RecursionsSelf/RecPlotF_h05_s02_Self.pdf", plotF4S];
Export["Figures_Jan2025/RecursionsSelf/RecPlotF_h05_s02_Self.jpg", plotF4S];
```

Section B: Gene conversion lengths, conditioning on non-zero tracts

The gene conversion (GC) routine works as follows:
- A ‘centre’ is defined for the GC event
- Two GC tracts are drawn going left, right from this centre from a geometric distribution with mean $\lambda/2$
- the total sum is hence from a negative binomial distribution.

The PDF of a neg bin distribution(n, p) with n = 1 is the same as a Geometric distribution in *Mathematica*:

In[•]:=
```
PDF[NegativeBinomialDistribution[1, p], x]
```

Out[•]=

$$\begin{cases} (1-p)^x p & x \geq 0 \\ 0 & \text{True} \end{cases}$$

In[ ]:=
```
PDF[GeometricDistribution[p], x]
```
Out[ ]=
$$\begin{cases} (1-p)^x p & x \geq 0 \\ 0 & \text{True} \end{cases}$$

And the mean of the geometric distribution is $\frac{1}{p} - 1$, so one is drawing for the number of *failures* before a success. This is the same definition that SLiM uses.

In[ ]:=
```
Mean[GeometricDistribution[p]]
```
Out[ ]=
$$-1 + \frac{1}{p}$$

So the total length is drawn from NegBin(2,p).

We want to know:
1) What is the distribution, *conditional on rejecting zero-length tracts?*
2) What is the *mean* of this conditional distribution?
3) How do we parameterize it in the simulations?

To answer (1), we use Bayes' theorem:

$P(X{=}x|X{!=}0) = \frac{P(X\neq 0 \mid X=x)\,P(X=x)}{P(X\neq 0)}$

P(X!=0|X=x) = 1 by logic.
P(X=x) is the negative binomial distribution, NB(2,p):

In[ ]:=
```
PDF[NegativeBinomialDistribution[2, p], x]
```
Out[ ]=
$$\begin{cases} (1-p)^x p^2 (1+x) & x \geq 0 \\ 0 & \text{True} \end{cases}$$

P(X!=0) can be calculated from the distribution as 1-P(X=0)

In[ ]:=
```
1 - PDF[NegativeBinomialDistribution[2, p], 0]
```
Out[ ]=
$$1 - p^2$$

So the conditional distribution is simply:

In[ ]:=
$$\frac{(1-p)^x p^2 (1+x)}{1-p^2}$$
Out[ ]=
$$\frac{(1-p)^x p^2 (1+x)}{1-p^2}$$

Checking that it is valid (i.e. it sums to 1)

In[ ]:=
$$\text{Sum}\left[\frac{(1-p)^x p^2 (1+x)}{1-p^2}, \{x, 1, \infty\}\right] \text{ // Simplify}$$
Out[ ]=
$$1$$

(2) What is the mean? It is simply this sum:

In[ ]:= $\text{Sum}\left[x\,\frac{(1-p)^x\,p^2\,(1+x)}{1-p^2},\ \{x,1,\infty\}\right]$

$$\frac{2}{p\,(1+p)}$$

(3) How do we parameterize this distribution?

Let y be the mean value we want to set our simulation to (e.g. 2000bp, denoted λ in the manuscript). Then $y=\frac{2}{p\,(1+p)}$.

Solving for p:

In[ ]:= $\text{Solve}\left[y == \frac{2}{p\,(1+p)},\ p\right]$

Out[ ]=

$$\left\{\left\{p\to\frac{-y-\sqrt{y}\,\sqrt{8+y}}{2\,y}\right\},\ \left\{p\to\frac{-y+\sqrt{y}\,\sqrt{8+y}}{2\,y}\right\}\right\}$$

Hence there are two possible solutions of p, but with some inspection it seems that only the latter yield valid values of p between 0 and 1. We can see this, for example, by taking the limit of both solutions between y = 1 (the lowest value) and infinity:

In[ ]:= $\text{Limit}\left[\left\{\left\{\frac{-y-\sqrt{y}\,\sqrt{8+y}}{2\,y}\right\},\ \left\{\frac{-y+\sqrt{y}\,\sqrt{8+y}}{2\,y}\right\}\right\},\ y\to 1\right]$

$\text{Limit}\left[\left\{\left\{\frac{-y-\sqrt{y}\,\sqrt{8+y}}{2\,y}\right\},\ \left\{\frac{-y+\sqrt{y}\,\sqrt{8+y}}{2\,y}\right\}\right\},\ y\to \infty\right]$

Out[ ]=

$\{\{-2\},\{1\}\}$

Out[ ]=

$\{\{-1\},\{0\}\}$

So when the geometric draws are defined in SLiM, if each is parameterized with $p=\frac{-y+\sqrt{y}\,\sqrt{8+y}}{2\,y}$ and zero-length tracts are discarded then the mean of the final distribution will equal y.

We can also calculate the CDF of this conditional distribution:

In[ ]:= $\text{Sum}\left[\frac{(1-p)^x\,p^2\,(1+x)}{1-p^2},\ \{x,1,z\}\right]$ // Simplify

Out[ ]=

$$-\frac{-1+(1-p)^z+p\,\left(-1+(1-p)^z+(1-p)^z\,z\right)}{1+p}$$

Then if we substitute $p=\frac{-y+\sqrt{y}\,\sqrt{8+y}}{2\,y}$ to define this CDF with overall mean y:

In[ ]:= $-\frac{-1+(1-p)^z+p\,\left(-1+(1-p)^z+(1-p)^z\,z\right)}{1+p}$ /. $p\to\frac{-y+\sqrt{y}\,\sqrt{8+y}}{2\,y}$ // Simplify

Out[ ]=

$$\frac{\sqrt{y}\left(1-\left(\frac{3}{2}-\frac{1}{2\sqrt{\frac{y}{8+y}}}\right)^z+\left(\frac{3}{2}-\frac{1}{2\sqrt{\frac{y}{8+y}}}\right)^z z\right)-\sqrt{8+y}\left(-1+\left(\frac{3}{2}-\frac{1}{2\sqrt{\frac{y}{8+y}}}\right)^z+\left(\frac{3}{2}-\frac{1}{2\sqrt{\frac{y}{8+y}}}\right)^z z\right)}{\sqrt{y}+\sqrt{8+y}}$$

In[ ]:= CDFCon[y_, z_] :=

$$\frac{\sqrt{y}\left(1-\left(\frac{3}{2}-\frac{1}{2\sqrt{\frac{y}{8+y}}}\right)^{z}+\left(\frac{3}{2}-\frac{1}{2\sqrt{\frac{y}{8+y}}}\right)^{z} z\right)-\sqrt{8+y}\left(-1+\left(\frac{3}{2}-\frac{1}{2\sqrt{\frac{y}{8+y}}}\right)^{z}+\left(\frac{3}{2}-\frac{1}{2\sqrt{\frac{y}{8+y}}}\right)^{z} z\right)}{\sqrt{y}+\sqrt{8+y}}$$

This function gives the probability that a draw will have less then length z, given mean y.
We can numerically solve it to work out what the upper quantile is for a given probability and overall mean.
For example, if y = 2000 then 99% of draws will be less than the following length:

In[ ]:= `NSolve[CDFCon[2000, z] == 0.99, z]`

NSolve: Inverse functions are being used by NSolve, so some solutions may not be found; use Reduce for complete solution information.

Out[ ]= `{{z → -998.305}, {z → 6640.17}}`

Obviously only the positive value is valid here, i.e. 6640.
If you want more events of longer tract length then we can use a larger mean. Letting y = 3000 means that 99% of events are less than the following:

In[ ]:= `NSolve[CDFCon[3000, z] == 0.99, z]`

NSolve: Inverse functions are being used by NSolve, so some solutions may not be found; use Reduce for complete solution information.

Out[ ]= `{{z → -1496.46}, {z → 9959.34}}`

So 1% of events exceed 9959 or nearly 10,000, so this mean might be better to use in the simulations. In this case, p would equal:

In[ ]:= $\frac{-y+\sqrt{y}\sqrt{8+y}}{2y}$ /. y → 3000 // N

Out[ ]= 0.000666223

In[ ]:= `NSolve[CDFCon[4000, z] == 0.95, z]`

NSolve: Inverse functions are being used by NSolve, so some solutions may not be found; use Reduce for complete solution information.

Out[ ]= `{{z → -1964.51}, {z → 9488.6}}`
